# Whole cell response to receptor stimulation involves many deep and distributed subcellular biochemical processes

**DOI:** 10.1016/j.jbc.2022.102325

**Published:** 2022-08-01

**Authors:** Jens Hansen, Mustafa M. Siddiq, Arjun Singh Yadaw, Rosa E. Tolentino, Vera Rabinovich, Gomathi Jayaraman, Mohit Raja Jain, Tong Liu, Hong Li, Yuguang Xiong, Joseph Goldfarb, Ravi Iyengar

**Affiliations:** 1 Department of Pharmacological Sciences and Institute for Systems Biomedicine, Icahn School of Medicine at Mount Sinai, New York, New York, USA; 2 Department of Microbiology, Biochemistry and Molecular Genetics, Rutgers University, New Jersey Medical School, Newark, New York, USA

**Keywords:** subcellular pathways, transcriptomics, bioinformatics, dynamical modeling, neurite outgrowth, CB1R, cannabinoid 1 receptor, DEG, differentially expressed gene, DEP, differentially expressed protein, DMSO, dimethyl sulfoxide, ER, endoplasmic reticulum, FDR, false discovery rate, MBCO, Molecular Biology of the Cell Ontology, MEM, minimum essential medium, NOG, neurite outgrowth, SCP, subcellular process, TGN, *trans*-Golgi network

## Abstract

Neurite outgrowth is an integrated whole cell response triggered by the cannabinoid-1 receptor. We sought to identify the many different biochemical pathways that contribute to this whole cell response. To understand underlying mechanisms, we identified subcellular processes (SCPs) composed of one or more biochemical pathways and their interactions required for this response. Differentially expressed genes and proteins were obtained from bulk transcriptomics and proteomic analysis of extracts from cells stimulated with a cannabinoid-1 receptor agonist. We used these differentially expressed genes and proteins to build networks of interacting SCPs by combining the expression data with prior pathway knowledge. From these SCP networks, we identified additional genes that when ablated, experimentally validated the SCP involvement in neurite outgrowth. Our experiments and informatics modeling allowed us to identify diverse SCPs such as those involved in pyrimidine metabolism, lipid biosynthesis, and mRNA splicing and stability, along with more predictable SCPs such as membrane vesicle transport and microtubule dynamics. We find that SCPs required for neurite outgrowth are widely distributed among many biochemical pathways required for constitutive cellular functions, several of which are termed ‘deep’, since they are distal to signaling pathways and the key SCPs directly involved in extension of the neurite. In contrast, ‘proximal’ SCPs are involved in microtubule growth and membrane vesicle transport dynamics required for neurite outgrowth. From these bioinformatics and dynamical models based on experimental data, we conclude that receptor-mediated regulation of subcellular functions for neurite outgrowth is both distributed, that is, involves many different biochemical pathways, and deep.

Neuronal differentiation that results in highly connected circuits responsible for brain function starts with the formation of multiple projections called neurites. Subsequently, these projections undergo further differentiation to become either axons or dendrites (1, 2). Neurite outgrowth is considered to be among the earliest steps in neuronal differentiation. It can be triggered by wide range of extracellular signals including netrins, growth factors, and ligands that regulate G protein–coupled receptors (GPCRs) (3, 4, 5). Regulation of neurite outgrowth (NOG) by GPCRs is often routed through the G_o/i_ pathway. We have studied cannabinoid 1 receptor (CB1R) regulated NOG as a model system for G protein–regulated whole cell responses and have shown that signal flow from G_o_ through Rap to Src and Stat3 control is required (6). A detailed analysis of the upstream signaling network in our laboratory demonstrated the key role of transcriptional regulation and the involvement of multiple transcription factors in CB1R-triggered NOG (7). We sought to identify the downstream subcellular mechanisms by which transcriptional control triggers NOG. Identifying the subcellular pathways involved could help elucidate the underlying subcellular mechanisms. To identify the transcriptionally regulated genes involved in early NOG, we conducted RNA-seq and proteomics experiments and identified the differentially expressed genes (DEGs) and proteins (DEPs) as the starting point for mapping downstream mechanisms.

The goal of this study was to obtain a genome-wide near-comprehensive description of the biochemical pathways involved in CB1R-regulated NOG in Neuro-2A cells and rat primary cortical neurons (8, 9, 10, 11, 12, 13). We had hypothesized that we might be able to track the relationships between the signaling network we had previously described (7) and the key cellular processes involved in NOG: microtubule growth and vesicle transport and fusion at the growth cone (14). However, inspection of the DEGs and DEPs by transcriptomics and proteomics analyses indicated that many diverse subcellular processes (SCPs) were likely to be involved. SCPs are one or more biochemical pathways consisting of multiple gene products, with a pathway having an input node and an output effector function that might be biochemical or physiological. How these pathways come together to trigger a whole cell response is not fully understood.

We have reasoned that individual pathways come together to form subnetworks that in turn form larger networks required for whole cell responses. To build such integrated networks, we identified SCPs and relationships between SCPs using standard cell biological knowledge found in textbooks such as Molecular Biology of the Cell (15), Biochemistry (16) and Medical Biochemistry (17) to develop a cell biological ontology called Molecular Biology of the Cell Ontology (MBCO) (18). This ontology allows us to associate genes/proteins with specific SCPs and specify the relationships among SCPs required for a whole cell function such as NOG.

We have analyzed the DEGs and proteins using MBCO and found various SCPs are likely to be involved, indicating that receptor regulation of NOG was likely to be a distributed phenomenon within the cell. We also found that many of the genes and proteins that were differentially expressed belonged to SCPs that could be considered generic, that is, required for functions in many cell types and often viewed as part of constitutive processes. Since such SCPs are distal from the signaling networks and the proximal functions such as microtubule growth and membrane vesicle transport, we call them deep SCPs. We used a combination of bioinformatics analyses and dynamical modeling to understand the relationships among the SCPs involved in NOG. To experimentally test the computational predictions regarding the SCPs involved and consequently the underlying mechanisms, we conducted siRNA ablation studies. We selected representative genes of the identified SCPs even if the selected genes themselves were not differentially expressed. Together, these experimental and computational analyses show that SCPs related to many diverse subcellular functions come together to mount an integrated whole cell response: NOG in response to CB1R stimulation. The organization of the interacting SCPs indicates that mechanism of NOG will be deep and distributed.

## Results

### Chronological activation of multiple distributed interdependent SCPs

**Figure 1:**
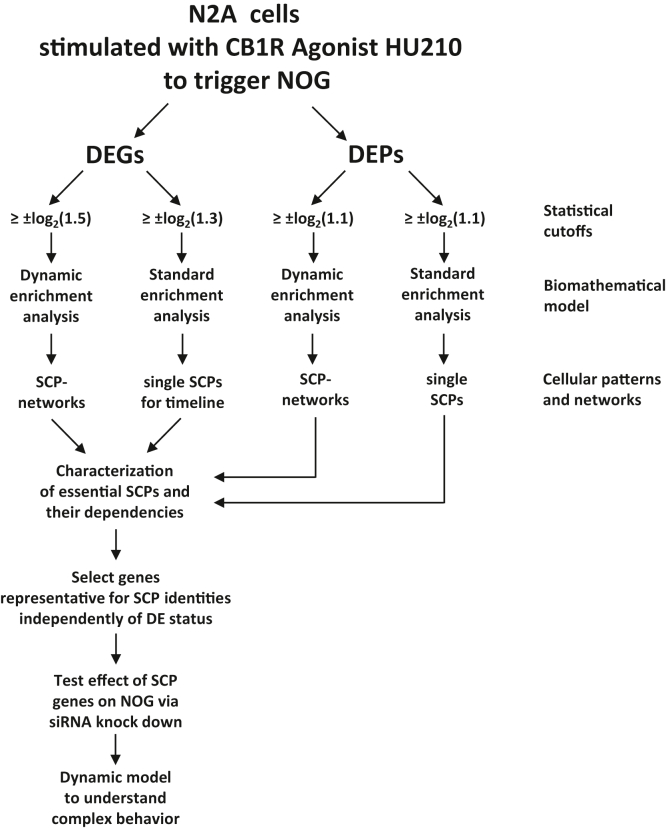
**Flow chart about data analysis and validation.** See main text for details.

To characterize the potential regulatory mechanisms that form the basis for an integrated whole cell response such as NOG, we stimulated the neuroblastoma cell line N2A with HU210, a potent activator of the CB1R (see Fig. 1 for flow chart). CB1R stimulation increases NOG, thereby offering a model system where a well-defined receptor can trigger a whole cell response. We stimulated N2A cells with HU210 for 2 h, 4 h, 6 h, and 8 h and investigated gene expression changes by bulk RNA-seq and for 5 h, 10 h, and 18 h and investigated protein expression changes by discovery proteomics. To identify canonical SCPs that enable NOG, we subjected the DEGs and DEPs to pathway enrichment analysis using the MBCO (18). MBCO SCPs are organized in 3 to 4 levels, where higher level SCPs (*i.e.*, levels with lower numbers) describe more general and lower level SCPs more detailed biological processes. The annotated vertical SCP hierarchy is enriched using a unique MBCO algorithm that infers horizontal relationships between functionally interacting SCPs of the same level. These relationships allow a new enrichment algorithm, dynamic enrichment analysis. In contrast to standard enrichment analysis, dynamic enrichment analysis does not only test if genes of interest are enriched among the genes annotated to single SCPs but also to combinations of functionally interacting SCPs. This computational operation allows us to develop context-specific SCP networks. Results of standard enrichment analysis were integrated into timelines that document the predicted activities of identified SCPs over the whole stimulation period. From our predictions, we characterized SCPs that are essential for NOG. To validate our findings, we selected representative SCP genes (independently of their differential expression status) and tested their effect on NOG *via* siRNA knockdown studies in primary rat cortical neurons.

To characterize the main drivers of cellular change of state, we initially subjected all DEGs with a minimum log_2_(fold change) of ±log_2_(1.5) to dynamic enrichment analysis. To obtain a more comprehensive view on changes involved in NOG, we also subjected DEGs with a less stringent cutoff (±log_2_(1.3)) to standard enrichment analysis. Our systems biology approach assumes that small changes in many functionally interacting genes (as defined by their annotation to the same pathway) can have a significant effect on the overall pathway activity.

**Figure 2:**
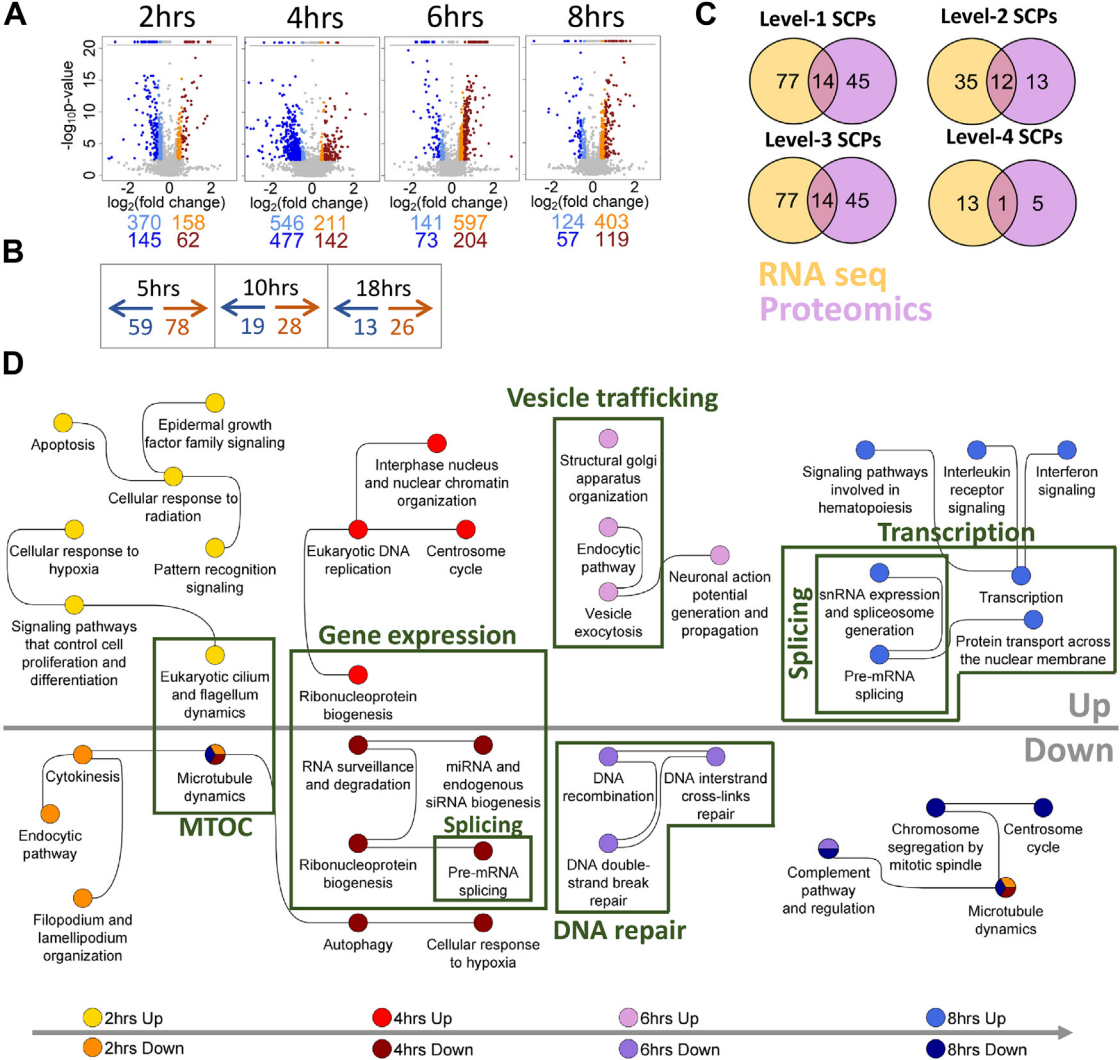

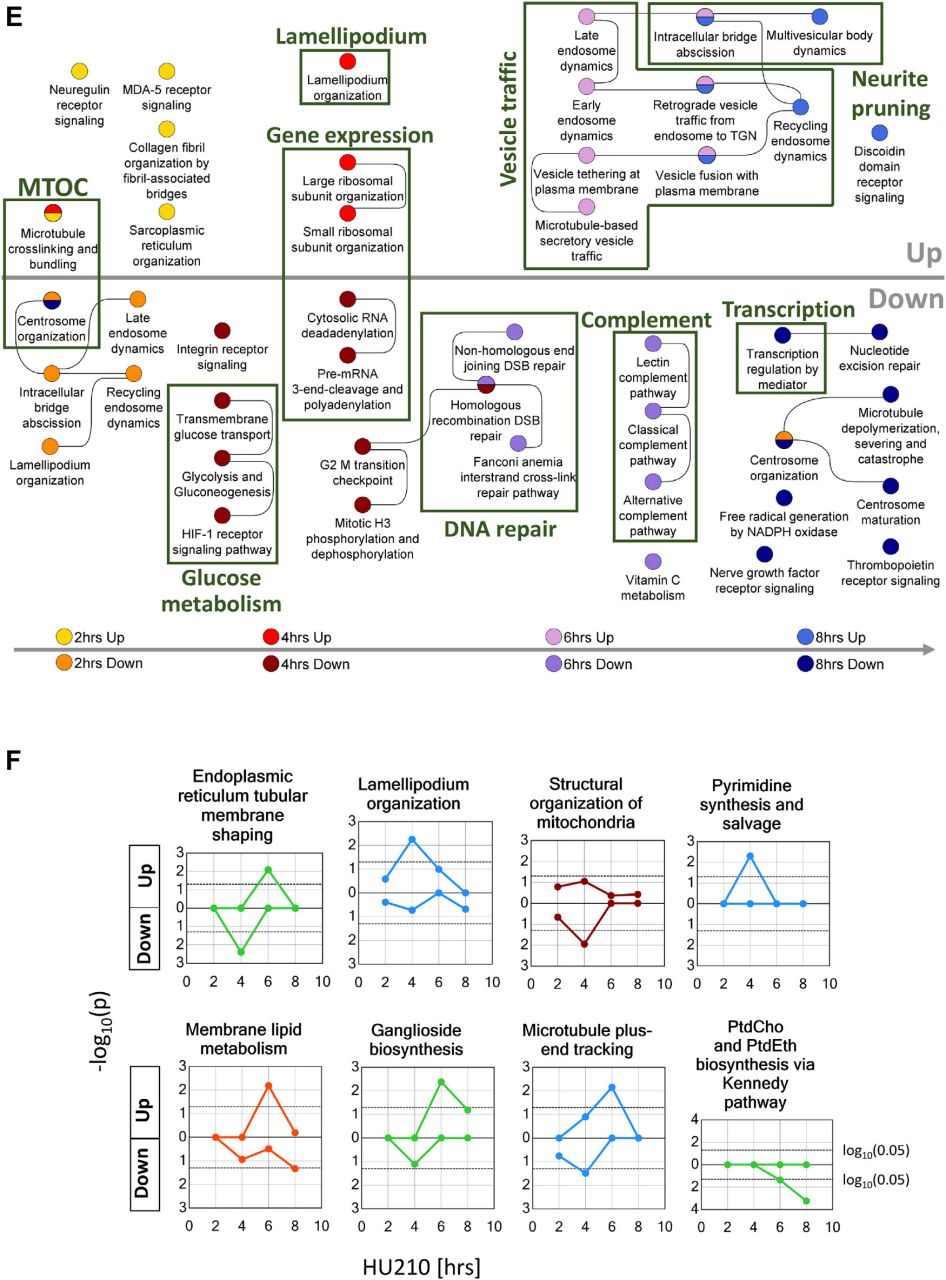
**Chronological activation of distributed interdependent SCPs during neurite outgrowth (NOG) matches their dependencies.***A*, Neuro2A cells were stimulated with the CB1R agonist HU210 for indicated time periods, followed by identification of differentially expressed genes (DEGs, FDR 5%). Upregulated and downregulated genes are in *orange* and *light blue*, respectively, if the absolute log_2_(fold change) is at least log_2_(1.3) and less than log_2_(1.5) or in *red* and *blue*, if the absolute log_2_(fold change) is larger than or equal to log_2_(1.5). *B*, differentially expressed proteins (DEPs) (nominal *p*-value 0.05, minimum log_2_(fold change) = ±log_2_(1.1)) were identified after 5 h, 10 h, and 18 h HU210 treatment of N2A cells. *C*, upregulated and downregulated genes and proteins were subjected to dynamic and standard enrichment analysis using the Molecular Biology of the Cell Ontology (MBCO). Venn diagram documents how many level-1, level-2, level-3, and level-4 SCPs were simultaneously identified by both technologies at least one time point, independently of the direction of change. Level-2 (*D*) and level-3 (*E*) SCPs predicted by dynamic enrichment analysis of upregulated and downregulated genes at each time point were integrated into shown SCP networks. *F*, timelines labeled with ‘Up’ and ‘Down’ document -log_10_*p*-values obtained for selected SCPs predicted from upregulated and downregulated genes, respectively. *Dotted lines* indicate the *p*-value significance cutoff (-log_10_(0.05)). See Figure S1 for all timelines predicted based on standard enrichment analysis. CB1R, cannabinoid 1 receptor; FDR, false discovery rate; SCP, subcellular process.

We identified 1261 and 1314 genes that were differentially expressed at least one time point based on the higher or lower log_2_(fold change) cutoffs, respectively (Fig. 2*A*, Table S1). Proteomic analysis revealed 214 proteins that were differentially expressed at least one time point (Fig. 2*B*, Table S2). Subjecting DEGs and DEPs to dynamic and standard enrichment analysis identified 166 (Fig. 2*C*, Tables S3 and S4) and 96 (Tables S5 and S6) level-1/-2/-3/-4 SCPs, respectively. Thirty-two of those SCPs were identified by both assays at atleast 1 time point.

Dynamic enrichment analysis of DEGs allowed us to relate time-dependent gene expression profiles to different NOG stages (Table S3). At the earliest time point at which we conducted transcriptomics, we found that receptor stimulation regulated genes that are involved in microtubule and centrosome dynamics (Fig. 2, *D* and *E* for level-2/3 SCP-networks, respectively). These dynamics are consistent with an association of the localization of the centrosome to the hillock of the future axon, an event that occurs at an early stage of NOG (19, 20, 21).

At 4 h, we observed gene expression pattern changes consistent with changes in activity of many biochemical pathways involved in multiple SCPs. Dynamic enrichment analysis of DEGs at 4 h predicted upregulation of gene expression in general (Fig. 2, *D* and *E*, Table S3). This prediction is based on the upregulation of ribosomal proteins and downregulation of CCR4-NOT complex (22) subunits that are involved in bulk mRNA degradation (23) and polymerase II suppression (24) (Fig. S4*A*). Suggested increased gene expression activity is additionally supported by downregulation of genes that stabilize the tubular endoplasmic reticulum (ER), indicating an increase in the amount of granular ER involved in membrane protein translation (Fig. 2*F*, Table S4) (25, 26). Predicted upregulation of lamellipodia formation is in agreement with morphological observations that neurites emerge from lamellipodia that form at the earliest stages of NOG (27). Activity changes in mitochondrial processes at the same time indicate mitochondria also prepare for outgrowth. These changes are consistent with prior observations that mitochondria accumulate at the hillock of the future axon before its major growth phase (28, 29). Upregulation of the pyrimidine synthesis and salvage pathway at 4 h can support gene expression because they generate the building blocks for mRNA synthesis and also support the notion that the cell is preparing for an increase in lipid membrane production, since the pyrimidine nucleoside triphosphates CTP and UTP are cofactors during membrane lipid synthesis (30).

At 6 h time point, we observe expression patterns consistent with key SCPs required for the main growth stage of NOG (Stages 2/3) (31). This phase is characterized by synthesis of massive amounts of membranes in the cell body that are transported to the neurite tip *via* microtubule-based vesicle transport. At the tip, the newly synthesized membrane is added to the neurite, enabling its elongation (32, 33). In agreement with this morphological observation, standard enrichment analysis of upregulated genes predicts the increased synthesis of membrane lipids, in particular gangliosides (Fig. 2*F*, Table S4) that constitute 10% to 20% of all lipids in neuronal membranes (34) and are enriched in the outer membrane leaflet of the neurite plasma membrane (35). Genes involved in stabilization of the tubular ER are upregulated at this time point. A tubular ER network spans the whole axon and is involved in multiple functions, including lipid biosynthesis, glucose homeostasis, and calcium supply (36). Our results suggest that generation of this network starts in parallel with initial neurite growth. Dynamic enrichment analysis predicts networks of interacting SCPs that are involved in membrane vesicle trafficking between the *trans*-Golgi network (TGN) and neurite tip that utilizes the microtubule tracts for movement (Fig. 2, *D* and *E*, Table S3). Consistently with this interdependency, we observed upregulation of a microtubule plus-end growth pathway (Fig. 2*F*, Table S4). Eight hours DEGs are enriched for multiple SCPs and SCP networks involved in further modification of vesicle trafficking, membrane lipid synthesis, as well as transcription and mRNA splicing (Fig. 2, *D–F*, Tables S3 and S4). Timelines of all SCPs predicted based on standard enrichment analysis are shown in Figure S1.

### Proteomic support of inferences from transcriptomic data

Enrichment analysis of DEPs confirmed many of the SCPs identified based on gene expression changes. Dynamic and standard enrichment analysis of DEPs after 5 h HU210 treatment predicted upregulation and downregulation of SCPs involved in gene expression, including mRNA splicing (Fig. S2*A*, Table S5). Microtubule dynamics were among the predicted upregulated SCPs by both enrichment approaches as well (Fig. S2, *A–C*, Tables S5 and S6). At later time points (10 h and 18 h), only a few proteins were differentially expressed (Fig. 2*B*) that were involved in multiple SCPs among which are SCPs related to gene expression and microtubule growth (Fig. S2 *A*, *B*, *D*, *E*, Tables S5 and S6).

### Experimental validation of predicted SCPs

To document the involvement of the predicted SCPs in NOG, we selected representative genes for siRNA knockdown in primary rat cortical neurons. Our aim was not to analyze if a certain gene is involved in a particular SCP but if a particular SCP is involved in NOG. Therefore, when possible, we selected genes whose knockdown has been shown to influence the corresponding SCP in other cell types, independent of its transcriptional regulation during NOG. Indeed, most of the genes selected for siRNA knockdown were not identified as DEGs or DEPs (Table 1).

**Table 1:** Selected genes for siRNA knockdown screening

Subcellular function	NCBI symbol	Is differentially expressed gene (DEG) or protein (DEP)?	NCIB description
Exosome complex mediated degradation of deadenylated mRNA	Dis3	No DEG/DEP	DIS3 homolog, exosome endoribonuclease and 3′-5′ exoribonuclease
mRNA splicing	Mbnl1	No DEG, Upregulated protein at 5h	muscleblind like splicing factor 1
mRNA splicing	Sf3a2	No DEG/DEP	splicing factor 3a, subunit 2
mRNA splicing	Snrnp70	No DEG/DEP	small nuclear ribonucleoprotein 70 (U1)
Pyrimidine *de novo* synthesis	Cad	No DEG/DEP	carbamoyl-phosphate synthetase 2, aspartate transcarbamylase, and dihydroorotase
Pyrimidine *de novo* synthesis	Dhodh	Upregulated gene at 4 h, no DEP	dihydroorotate dehydrogenase
Pyrimidine salvage	Tymp	Upregulated gene at 4 h/NO DEP	thymidine phosphorylase
Sphingomyelin synthesis	Col4a3bp	No DEG/DEP	collagen, type IV, alpha 3 (Goodpasture antigen) binding protein
Synthesis of complex gangliosides	Plekha8	No DEG/DEP	pleckstrin homology domain containing, family A (phosphoinositide binding specific) member 8
Synthesis of complex gangliosides	Ugcg	Downregulated gene at 2 h, Upregulated gene at 6 h and 8 h, no DEP	UDP-glucose ceramide glucosyltransferase
Phosphatidylcholine (PC) synthesis	Pcyt1a	No DEG/DEP	phosphate cytidylyltransferase 1, choline, alpha isoform
Phosphatidylethanolamine (PE) synthesis	Pcyt2	No DEG/DEP	phosphate cytidylyltransferase 2, ethanolamine
Cotranslational protein insertion into ER	Srp54a	No DEG/DEP	signal recognition particle 54A
Mitochondrial transport	Rhot1	Downregulated gene at 4 h, no DEP	ras homolog family member T1
Mitochondrial fusion	Mfn2	No DEG/DEP	mitofusin 2
Mitochondrial translation	Tufm	No DEG/DEP	Tu translation elongation factor, mitochondrial
Mitochondrial protein import	Mtx1	Downregulated gene at 6 h, Upregulated protein at 5 h	metaxin 1
Golgi derived vesicle loading on microtubules, actin-microtubule crosslinking	Macf1	Downregulated gene at 4 h, Upregulated gene at 6 h, no DEP	microtubule-actin crosslinking factor 1
Vesicle priming during exocytosis	Unc13b	No DEG/DEP	unc-13 homolog B
Exocyst-mediated vesicle tethering during exocytosis	Exoc4	No DEG/DEP	exocyst complex component 4
Exocyst-mediated vesicle tethering during exocytosis	Expc7	No DEG, Upregulated protein at 5 h	exocyst complex component 7
Clathrin-mediated endocytosis	Ap2m1	No DEG/DEP	adapter related protein complex 2 subunit mu1
Retromer-mediated endosomal sorting	Snx2	No DEG/DEP	sorting nexin 2
Retromer-mediated endosomal sorting	Vps35	No DEG/DEP	VPS35 retromer complex component
Microtubule plus-end growth	Mapre1	Upregulated gene at 6 h, Upregulated protein at 5h	microtubule-associated protein, RP/EB family, member 1
Cytokinesis/neurite pruning/multivesicular body formation	Chmp6	No DEG, Downregulated protein at 5 h	charged multivesicular body protein 6
Multivesicular body formation	Hgs	No DEG/DEP	HGF-regulated tyrosine kinase substrate
Cytokinesis/neurite pruning/multivesicular body formation	Tsg101	No DEG/DEP	tumor susceptibility gene 101
Tubular endoplasmic reticulum formation	Atl2	No DEG/DEP	atlastin GTPase 2
Tubular endoplasmic reticulum formation	Rtn1	No DEG/DEP	reticulon 1
Complement activation	Masp2	Downregulated gene at 4 h and 6 h, no DEP	mannan-binding lectin serine peptidase 2
Copper transmembrane transport	Atp7b	No DEG/DEP	ATPase, Cu++ transporting, beta polypeptide
Insulin mediated glucose import	Slc2a4	No DEG/DEP	solute carrier family 2 (facilitated glucose transporter), member 4
N-glycan maturation	Man1a2	Upregulated gene at 6 h, Upregulated protein at 5 h	mannosidase, alpha, class 1A, member 2
Synthesis of non-ATP Nucleoside triphosphates	Nme1	Upregulated gene at 2 h and 4 h, no DEP	NME/NM23 nucleoside diphosphate kinase 1
Nuclear protein translocation	Nup88	Upregulated gene at 8 h, no DEP	nucleoporin 88

We selected representative genes for the indicated subcellular functions to test the effect of their knockdown on neurite outgrowth. Most of the selected genes were not among the differentially expressed genes (No DEG) or differentially expressed proteins (No DEP) after HU210 treatment of N2A cells.

**Figure 3:**
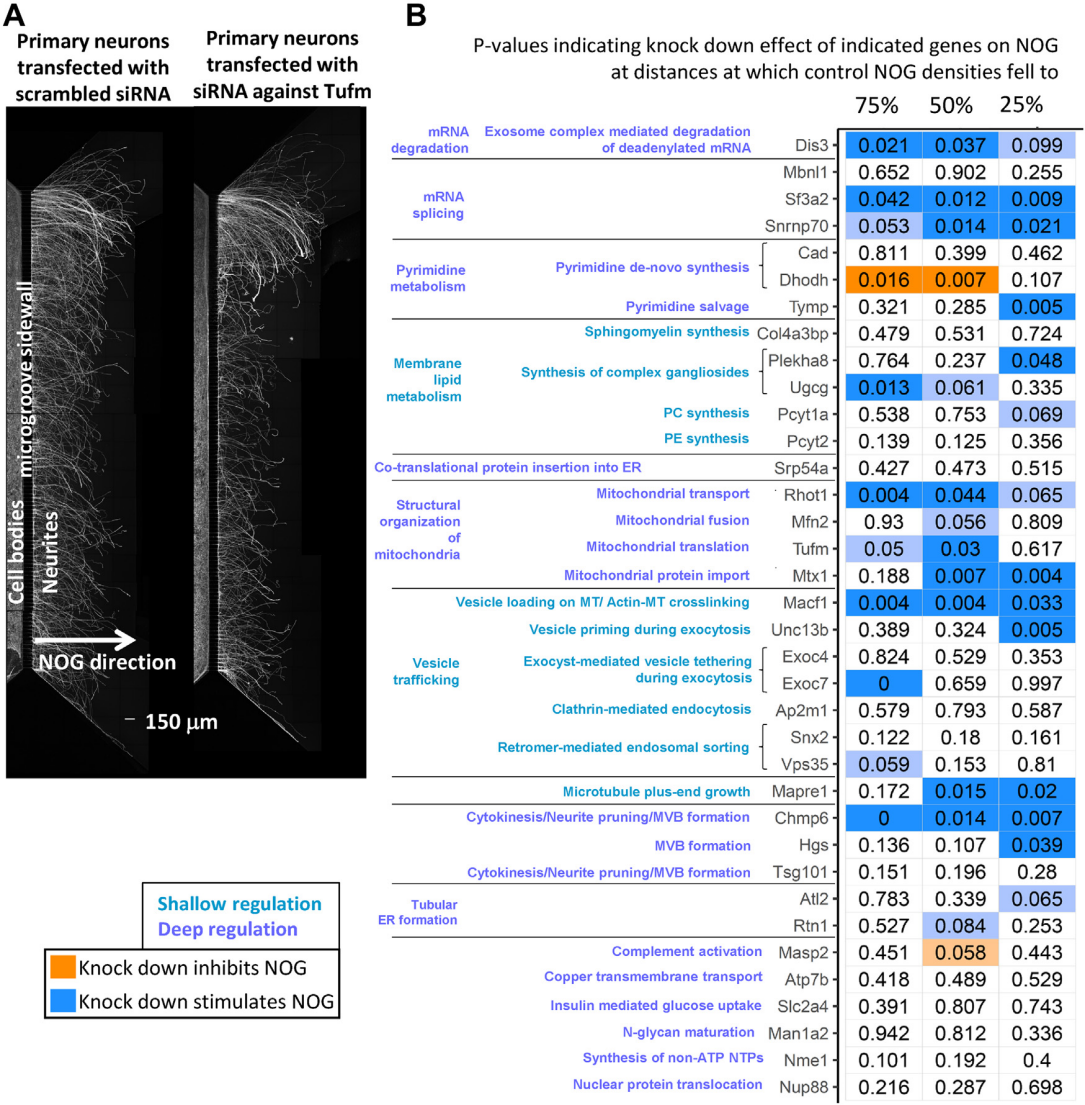
**siRNA knockdown experiments document involvement of multiple SCPs.***A*, P1 rat cortical neurons were transfected with siRNA against selected representative SCP genes (shown: Tufm) or scrambled siRNAs. To analyze neurite growth under gene silencing conditions we waited for 48 h after transfection, before resetting neurite growth to the edge of the microgroove via axotomy. Another 48 h later, we stained neurites and cell bodies for β-III tubulin to document siRNA effect on neurite outgrowth. Each shown image is a composite of three different overlapping images that showed the top, middle and bottom part of the microchamber and were manually stitched. *B*, neurite outgrowth fluorescence intensities were quantified and the average intensity at each distance from the microgroove sidewall calculated. All intensities of one experiment were normalized toward control average intensity at 100 μm that was set to 100%. Since outgrowth results showed great variations between the different experiments, we searched for those distances at which control average intensities had fallen to 75%, 50%, and 25%. We compared the outgrowth intensities between siRNA-treated and control samples at these three distances. Nominal *p*-values are shown and labeled *dark blue* or *dark orange*, if knockdown significantly inhibited or stimulated neurite outgrowth (*p*-value ≤ 0.05), respectively, and *light blue* or *light orange*, if knockdown may inhibit or stimulate NOG (*p*-value > 0.05, ≤ 0.1), respectively. ER, endoplasmic reticulum; MT, microtubule; MVB, multivesicular bodies; PC, phosphatidylcholine; PE, phosphatidylethanolamine; SCP, subcellular process. See Figure S3*D* for detailed results.

Primary rat cortical neurons were seeded on microfluidic chambers that contain two sides separated by a microgroove sidewall (Fig. 3*A*). Plated on the left side of the chamber, the cell bodies cannot cross to the other side, but their neurites can grow through small slits in the sidewall into the right side, allowing an easy quantification of outgrowth length. After siRNA transfection, we waited for 48 h to allow siRNA-mediated gene silencing and performed axotomy to reset neurite growth length in all chambers to zero (*i.e.*, to the microfluidic sidewall). Another 48 h later, we microscopically documented NOG in each chamber, performed quality control (Fig. S3, *A–C* and Table S7), and analyzed siRNA knockdown effects on NOG. Average growth densities showed a steady decrease along the outgrowth direction. In each experiment, we compared the average growth densities between knockdown and control cells at three different locations: we selected those distances at which the control densities had fallen to 75%, 50%, and 25% from the initial density measured at 100 μm distance from the wall. Any gene knockdown that showed a nominally significant change of growth density (α = 0.05) at least one of these three distances was defined to significantly regulate NOG. In a few cases where significance was closely missed (*p*-value > 0.05 and ≤ 0.1), we stated that the gene may influence NOG.

We could verify the involvement of multiple SCPs that we identified in our RNA-seq screening approach (Fig. 3*B*, Fig. S3*D*). Among these were SCPs that can be directly linked to neurite shaft growth, that is, membrane lipid production, membrane delivery to the growth cone *via* vesicle trafficking, microtubule plus-end growth and neurite pruning, or multiple vesicular body formation. Among the validated SCPs that describe deep regulatory functions that contribute to NOG on a lower more basal level were mRNA splicing, pyrimidine synthesis and salvage, SCPs involved in mitochondrial dynamics, and tubular ER formation. In total, we tested 38 different genes for their effect on NOG. Knockdown of 16 siRNAs had a significant effect on NOG, while knockdown of another 6 genes may influence NOG. In a few cases, genes were selected to distinguish between two different subpathways involved in the same function. For example, we selected the two genes Cad and Dhodh and the gene Tymp to distinguish between pyrimidine *de novo* synthesis and pyrimidine salvage, respectively (37). The gene Hgs is a component of the ESCRT-0 complex that is involved in multivesicular body formation but not in cytokinesis or neurite pruning (38). The gene was selected to distinguish between these two pathways, though an effect of its knockdown might also be due to its involvement in additional functions, for example, actin-mediated endosomal sorting (39). Similarly, the lipid transport genes Plekha8 and Col4a3bp that direct glycosylceramides to sites of ganglioside or sphingomyelin synthesis, respectively (40, 41, 42), were selected to distinguish between those two pathways. As can be seen, we did not select the genes with the intention to identify a maximum number of genes influencing NOG but anticipated characterization of genes with no influence on NOG as well. In other cases, redundant gene functions, for example, between the tested gene Snx2 and the not tested gene Snx1 (43) might explain missing documentation of knockdown effects on NOG. In summary, the experimental results agree with the effect that we expected by integrating the results of our transcriptomic and proteomic analysis with prior knowledge for 27 of 36 tested siRNA knockdowns (Table S8). For detailed integration of the gene expression data with the siRNA knockdown results, see ‘Further descriptive analyses of knockdown results’ in the supplemental information and Figure S4. Here, we want to highlight two findings.

### Increased forward growth depends on increased membrane back transport from the growth cone to the cell body

DEGs induced by 6 h and 8 h HU210 treatment are related to multiple steps in vesicle transport between the plasma membrane, endosome, and TGN (Fig. 2, *D* and *E*). Our siRNA screening supported this observation, since we could document that multiple SCPs ranging from vesicle loading on the microtubule after budding at the TGN (44), vesicle exocytosis, and retromer-based endosomal sorting (45, 46) are involved in NOG (Figs. 3*B* and S3*D*). Interestingly, SCP networks at 6 h and 8 h (Fig. 2*E*) also contained the SCP ‘retrograde vesicle traffic from the endosome to the TGN’ that describes membrane back transport from the growth cone to the TGN. Knockdown of the gene Vps35 that, as a major component of the retromer, participates in protein sorting from the endosome to the plasma membrane or TGN (45, 46) tends to inhibit NOG. Knockdown of the retromer component Snx2 has no effect on NOG, probably due to its functional redundancy with Snx1 (43).

**Figure 4:**
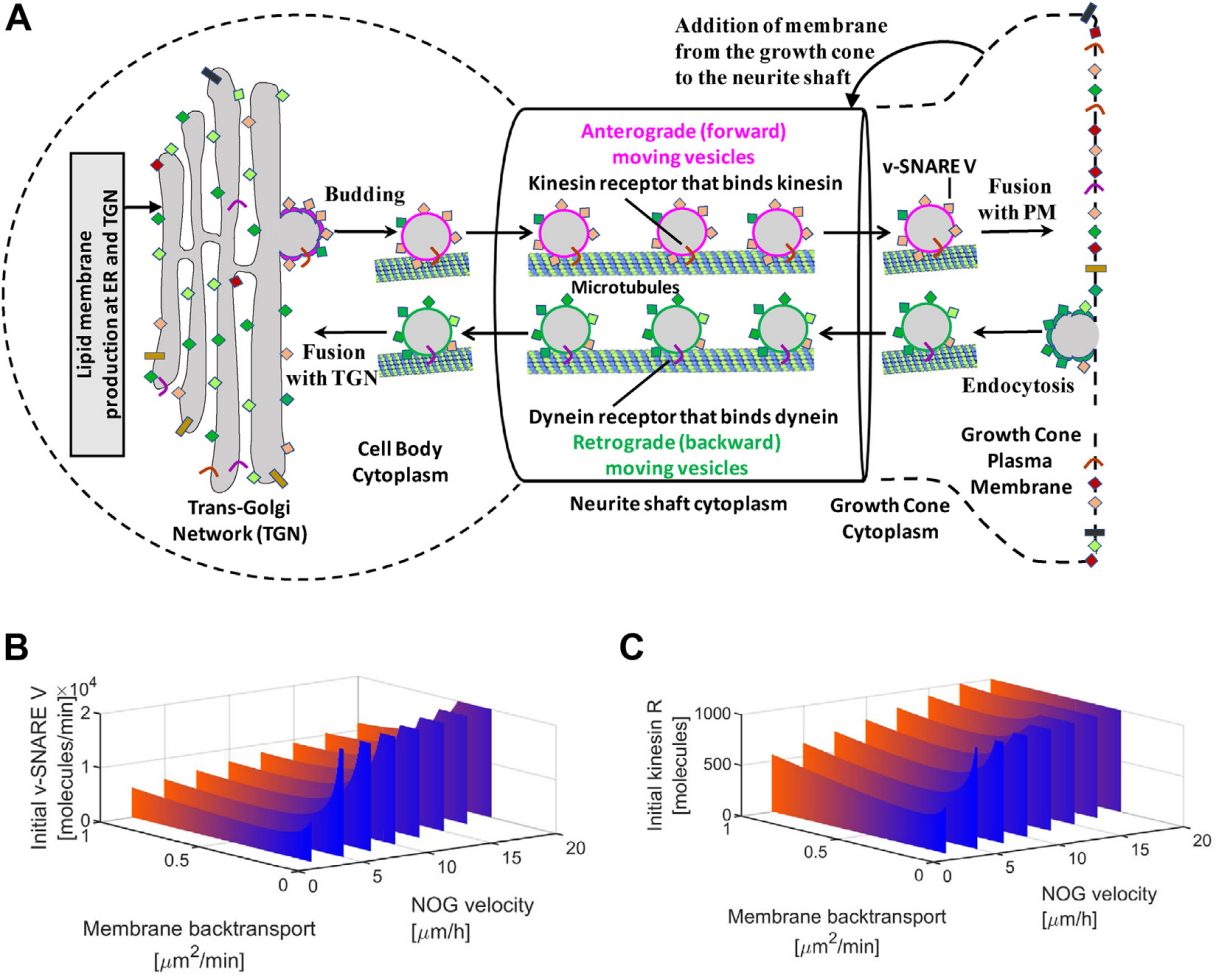
**Dynamic model of vesicular transport predicts that an increase in vesicle back transport is necessary for sufficient back transport of vesicle(v)-SNAREs and kinesin receptors from the growth cone to the TGN.***A*, we extended our compartmental ordinary differential equation model that simulates membrane production, vesicle membrane transport to the growth cone, and microtubule growth to analyze how membrane back transport influences NOG (see main text and experimental procedures for details). Part of our NOG model is an analytical solution that predicts kinetic parameter sets that allow NOG growth under a certain velocity without violation of SCP model constraints. Figure adapted from (47). Our analytical solution predicts that for each velocity there is a threshold for the membrane back transport rate below which the amounts of (*B*) v-SNAREs and (*C*) kinesin receptors rapidly increase. Below a second threshold, NOG is no longer possible without violation of the model constraints (lack of entries past the right front edge of each wall). Both thresholds shift to higher rates with increasing NOG velocity. See main text for details. NOG, neurite outgrowth; SCP, subcellular process; TGN, *trans*-Golgi network.

For investigation of this counter-intuitive finding that upregulated membrane transport from the growth cone to the cell body is a requirement for increased forward growth, we extended our multicompartment ordinary differential equation (ODE)-based NOG model (47). This model integrates membrane production, membrane delivery to the growth cone *via* vesicle transport, and microtubule growth during NOG. It simulates anterograde (forward) and retrograde (backward) vesicle trafficking between the TGN and the growth cone (Fig. 4*A*). Anterograde vesicles bud at the TGN and move through the neurite shaft to the growth cone cytoplasm *via* kinesin-mediated transport along the microtubules. Kinesin is attached to the vesicles *via* kinesin receptors that are integral parts of the vesicle membrane. Anterograde vesicles within the growth cone cytoplasm fuse with the growth cone plasma membrane after SNARE complex formation between vesicle(v)-SNAREs V and target(t)-SNAREs at the plasma membrane. Any additional membrane at the growth cone is added to the neurite shaft, causing its growth. Retrograde vesicles are generated *via* endocytosis at the growth cone membrane, transported backward along the microtubules *via* dynein-mediated vesicle transport into the cell body cytoplasm, and fuse with the TGN. The growing neurite acts as a sink for vesicle proteins because a longer neurite will contain more trafficking vesicles. Consequently, our model does not only demand continuous production of lipid membrane but also of vesicle proteins, in agreement with our observation that before the major growth phase, the capacity for gene expression is upregulated. Since some but not all kinetic parameters are known, part of our NOG model is an analytical solution that predicts kinetic parameter sets allowing NOG growth at a certain velocity (an experimentally measured high-level constraint) without violation of additional literature-curated SCP model constraints.

Using our dynamic model, we predicted that an increase in NOG velocity demands an increase in the total number of vesicle(v)-SNAREs (Fig. 4*B*) and vesicle kinesin receptors (Fig. 4*C*) in the neuronal cell. The higher the outgrowth velocity the more vesicle membrane needs to be transported to the growth cone. Consequently, higher velocities demand more v-SNAREs and kinesin receptors to equip the anterograde moving vesicles. Our model also predicts that the amount of needed v-SNAREs and kinesin receptors increases exponentially if the back transport rate is below a certain threshold. Below a second lower threshold, it is no longer possible to achieve NOG without violation of the model constraints (no simulation output past the right front edges of the walls in Figure 4, *B* and *C*). Any v-SNAREs and kinesin receptors that are brought to the growth membrane need to be back transported to the TGN to be available for the next set of anterograde moving vesicles. The binding sites for v-SNAREs and kinesin receptors on a vesicle are limited. Consequently, to secure the back transport of more v-SNAREs and kinesin receptors at higher outgrowth velocities, more vesicles have to move backward, increasing the total membrane back transport rate. This is indicated by the upregulated genes involved in back transport (Snx3, Rab9, Rab6a, Sgsm2, Pafah1b1, Rab43) (Fig. S4*E*) and the knockdown (Vps35) results. Thus, dynamical models can give mechanistic insight into predictions obtained from omic datasets and validated *via* siRNA knockdown studies.

### Low abundancies of basal spliceosomal components might favor mRNA translation in the cell body, high abundancies mRNA transport to the neurite tip

We could confirm the predicted involvement of splicing in NOG by knockdown of the spliceosomal components Sf3a2 and Snrnp70. Knockdown of either gene significantly inhibited NOG (Fig. 3*B*, and S3*D*). Integration of the splicing activities that we predicted from the RNA-seq data with the chronological order of the other pathways initially seems to be counter intuitive. Components of the splicing machinery are not upregulated but downregulated after 4 h HU210 treatment (Fig. 2*D*), that is, at that time point where other enrichment results suggest upregulation of the capacity for translation to prepare for the major NOG growth phase. After 8 h HU210 treatment, that is, after the major growth phase, we predict upregulation of the splicing machinery in combination with upregulation of a second transcriptional wave. This chronological order might suggest that the concentration of basal splicing components contributes to the regulation of mRNA fate in the neuron. mRNA molecules synthesized in the nucleus can have multiple fates among which are translation in the cell body but also transport to the neurite tip. Axonal growth cones contain a reservoir of translationally silent mRNAs (48) and the machinery for local translation, protein folding, and posttranslational modification (49). More than half of the neurite-localized proteome might have been generated by local mRNA translation (50). Alternative splicing of 3′ UTRs localizes neuronal mRNA molecules to neurites (51). Any population of a neurite or axonal growth cone with mRNAs demands that there is a neurite, so it should happen at an advanced state, in our model system after the major growth phase at 6 h. Consequently, the time points at which we predict downregulated and upregulated splicing activities suggest that low concentrations of basic splicing components favor the generation of mRNA splice variants for translation in the cell body, while high concentrations favor variants subjected to axonal transport. In agreement with our hypothesis, it was documented in other model systems that restriction of spliceosomal components alters splicing of a subset of mammalian mRNAs with weak 5′ splice sites (52).

Nevertheless, we cannot exclude that the observed upregulation of basal splicing components at 8 h is involved in a different cell physiological process besides NOG. Our knockdown results document the importance of splicing for NOG, but do not allow any further conclusions about the contribution of splicing to the different outgrowth stages. The documentation of a downregulated splicing machinery before the major outgrowth phase does not imply that there is no splicing at all. Our knockdown experiments could simply reduce splicing to an extend exceeding that physiological downregulation and thereby inhibiting NOG, even before the major outgrowth phase. Follow-up experiments are needed to investigate our hypothesis that the abundancy of basal splicing components decides between mRNA translation in the cell body and mRNA transport to the neurite tip or axonal growth cone.

## Discussion

### Multiple constitutive pathways are regulated to drive NOG

Several previous studies in mice (53, 54) and in worms (55) have shown that during axonal regeneration after injury, DEGs encode components of numerous cell signaling pathways including those involved in synaptic plasticity as well as SCPs involved in vesicle transport (56, 57). A genome-wide screen during regeneration identified a distinct set of genes including members of the Socs family, inhibitors of signaling through Stat3. This finding is in agreement with our previous finding of an essential role for Stat3 in NOG (7). Another prominent gene identified in the genome-wide screen was Rab27, a protein involved in vesicle transport (53). We started with a different approach, where our goal was to broadly identify genes regulated by a GPCR, the CB1R, so that we could identify the SCPs involved and how they connected with one another to produce a whole cell function. Therefore, it was not entirely unexpected that these DEGs and DEPs are somewhat different from those previously reported, which focused on control of the NOG process.

**Figure 5:**
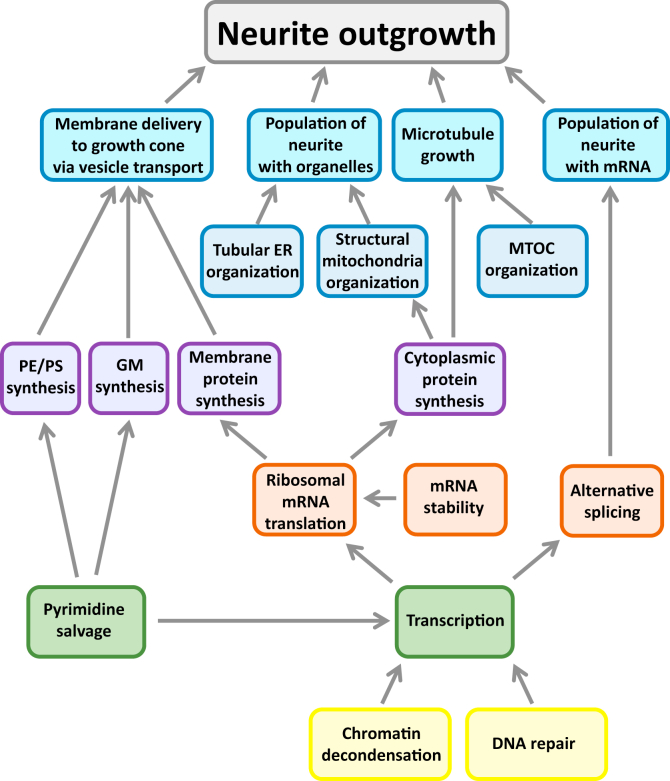
**Neurite outgrowth depends on deep and distributed regulation.** Multiple SCPs interact with each other to enable NOG. The SCPs describe subcellular functions that can either be directly associated with neurite shaft and neurite scaffold growth, for example, ‘Membrane lipid delivery to the growth cone’ and ‘Microtubule growth’, or describe deeper more basal subcellular functions, for example, ‘Pyrimidine salvage’. The network proposes SCP interactions that constitute the deep and distributed regulation of NOG. NOG, neurite outgrowth; SCP, subcellular process.

In looking through the SCPs and the DEGs and DEPs involved, it became clear that CB1R regulation of NOG is both deep and distributed (Fig. 5). By distributed, we mean that a wide range of SCPs are needed. By deep, we mean that SCPs involved are often part of general cellular function (constitutive pathways) and not necessarily selective for NOG or even neuronal functions. This includes regulation of pyrimidine metabolism, mitochondrial transport, and regulation of RNA stability. Regulation of these SCPs can affect many cellular responses in many different cell types, and hence, it is possible that such deep regulation may be a common feature of cell state change. Future experiments in other cell types will be needed to determine if these same SCPs are involved in other cell state change events.

### Validation of identified constitutive pathways

In order to validate the functional role of the SCPs inferred from DEGs, we used a strategy that is orthogonal to our initial screening approach in multiple parts. Our systems-biology-based identification of SCPs is based on the assumption that whole cell responses are driven by small accumulating changes in multiple gene products that participate in the same function. Consequently, we considered significantly changing DEGs and DEPs with relatively modest fold change cutoffs (±log_2_(1.3)/±log_2_(1.5) or ±log_2_(1.1), respectively). To identify cellular functions with many cumulative changes, we subjected the DEGs and DEPs to pathway enrichment analysis. In contrast, our validation approach targeted single SCP genes *via* siRNA knockdown that can cause an almost complete reduction in gene expression. Our strategy assumes that an almost complete (though not physiological) disruption in the expression of one representative SCP gene has a similar effect on SCP function as the modest reduction in the expression of multiple SCP genes. Thus, we used a reductionist approach to validate our systems level findings. In agreement with this line of reasoning, we did not only knock down SCP genes that were differentially expressed but also SCP genes, whose expression levels did not change. Besides this methodological difference, we conducted the siRNA-based knockdown studies using rat cortical neurons in primary culture without H210 treatment rather than the Neuro 2A cell line to test the general relevance of our line of reasoning. We also studied NOG in microfluidic chambers where we could sever initially growing neurites and then measure neurite growth in a standardized way. Although it was not our intent to study the SCPs involved in axonal regeneration after injury, our experimental approach allows us to reasonably suggest that the SCPs we identified could play a role in axonal regeneration.

### Study limitations

A limitation of this reductionist validation approach lies within gene pleiotropy. Most genes participate in multiple cellular functions that might all be altered by their knock downs. The genes that we selected for our knockdown studies participated in the same SCPs that were identified by the prior independent transcriptomic and proteomic analysis. Nevertheless, we cannot exclude that documented effects on NOG arise from the disturbance of different unconsidered cellular functions of those genes. As far as possible, we tried to address this concern by selecting two instead of one SCP genes to document the involvement of an SCP. Nevertheless, this was not always possible and sometimes only one of the selected genes had an effect.

Our validation approach allowed us to focus on SCPs rather than on individual genes. Overall, the siRNA-based knockdown experiments allowed us to validate a diverse set of SCPs required for NOG. It should be noted that the magnitude of the effects are modest, both for the differentially expressed genes as well as the effect of knockdown on NOG. This is not surprising given the wide range of SCPs involved. As documented for multifactorial diseases such as diabetes mellitus type II (58) or abdominal aortic aneurysm (59), individual genes only have small to moderate effects but interact with each other to enable the whole cell response.

## Conclusion

In conclusion, a combination of bioinformatics analyses and dynamic modeling enabled us to identify a broad range of SCPs that are required for receptor stimulation of NOG. Many of these SCPs are labeled deep as they appear to be constitutive SCPs that would be operational in many cell types. For many such deep SCPs, we were able to find previous independent studies that had individually implicated a role for the SCPs in NOG. These include phospholipid biosynthesis (60) and mitochondrial functions (61). Although our study provides strong suggestive evidence, our findings cannot be interpreted as definitive proof that the SCPs we describe here are involved in NOG in all conditions and in all species. Such proof will have come from future in-depth small-scale experiments that analyze the role of the SCP of interest in terms of the different genes involved. Even with this cautious interpretation, we have in this study described how a receptor can trigger a whole cell response by regulating a diverse set of biochemical pathways underling many SCPs and the relationship between these SCPs in enabling the whole cell response—NOG.

## Experimental and computational procedures

### Materials

Most reagents were purchased from Millipore Sigma, Fischer Scientific, or Illumina and were the highest available grade. For cell culture minimum essential medium (MEM), catalog #M4655, fetal bovine serum catalog # F4135, sodium pyruvate solution CAS No.: 113-24-6, and penn strep P4333 were used. For protein extraction, we used 8 M urea (Fisher catalog no.: #NCO128954) in 50 mM ammonium bicarbonate (Millipore Sigma, catalog no.: #09830-500 G). All other proteomics reagents were analytical grade and their use has been recently described (62). All materials for library preparation for bulk transcriptomic analyses reagents and kits were from Illumina. The Illumina stranded mRNA prep Ligation kit,catalog no.: #200040532, and index adapter, catalog no.: #20040554 were used. Microfluidic chambers were from Xona Microfluidics and Mattek dishes (catalog no.: #P50G-1.5) were from MatTek Corp.

### Cell culture

mRNA was prepared from 2 to 3 pooled independent experiments to achieve a sufficiently high amount of mRNA. Briefly, for each experiment, 1 million Neuro2A cells were seeded on each of eight 15 cm dishes and cultured for about 24 h in MEM (supplemented with 10% fetal bovine serum, 1 mM sodium pyruvate, and 1% penicillin/streptomycin). Low cell counts ensured low confluence leaving sufficient space for NOG. After 16 h starvation in serum-free MEM supplemented with 0.1% bovine serum albumin, we added HU210 (dissolved in dimethyl sulfoxide [DMSO]) to the media in a final concentration of 2 μM. Control cells were treated with equal volumes of drug-free DMSO. Cells were incubated for 2 h, 4 h, 6 h, and 8 h before replacement of the media by 8 ml TRIZOL, followed by collection and freezing of the cell suspension.

Cell pellets for proteomic analysis were prepared from 2 independent experiments. For each experiment, we plated 1 million Neuro 2A on each of seven 15 cm dishes and cultured them for about 24 h, followed by 1 h starvation. Media conditions were the same as described before. HU210 or DMSO was added as described before and cells were incubated for 5 h, 10 h, and 18 h, before stopping of the experiment by putting the cells on ice and replacing the media with ice-cold PBS. Cells were harvested and centrifuged (500*g*, 10 min), and cell pellets were shock frozen using liquid nitrogen. Untreated cell pellets were harvested directly after starvation.

### RNA-seq—sample preparation and sequencing

RNA was prepared using the standard TRIZOL protocol (TRIzol Reagent, Invitrogen). RNA was subjected to quality control *via* an Agilent Bioanalyzer. All samples of each experiment had an RNA integrity score of 10. Forty micrograms of total RNA was used for each sample. mRNA was purified from the RNA using a Dynabeads mRNA Purification Kit and fragmented *via* sonication using the Covaris E210. Complementary DNA libraries were prepared as described previously (63, 64, 65). Fragments of 200 to 300 base pairs length were selected *via* gel size selection. Complementary DNA libraries were subjected to sequencing using an Illumina HiSeq 2000 machine. Each sample was subjected to a single lane, yielding 117 to 128 million reads per sample.

### Identification of DEGs

Raw reads were aligned to the mouse reference genome mm9 using Tophat 2.0.8., samtools-0.1.7, and bowtie 2.1.0. Tophat was used with the ensemble GTF file as a gene annotation reference and the option 'no-novel-juncs'. Output BAM files were directly subjected to differential gene expression analysis using Cufflinks with the options 'multiread-correct', 'upper-quartile norm', and 'frag-bias-correct' against the mm9 genome. DEGs were identified based on adjusted *p*-value (false discovery rate [FDR] 5%) and a minimum fold change (log_2_[(FPKM_HU210_+1)/(FPKM_DMSO_ + 1)] >= ±log_2_(1.5) or ±log_2_(1.3)).

### Proteomics—sample preparation and mass spectrometry

Collected cell pellets were lysed in extraction buffer (8 M urea, 1% N-octyl glucoside, 100 mM triethylammonium bicarbonate, 1× protease inhibitor, 1× phosphatase inhibitors) and clarified by centrifugation. Proteins were then quantified using BioRad Bradford Protein Assay Kit with bovine serum albumin as standard. One hundred micrograms of proteins from each sample was digested overnight with trypsin at 37 °C. Samples were split into 2 different groups that were processed independently (group 1: 2× untreated cells, 2× 10 h HU210 and 2× 10 h DMSO treated cells and 2× 5 h HU210 treated cells, group 2: 2× untreated cells, 2× 18 h HU210, 2× 18 h DMSO and 2× 5 h DMSO treated cells). Both groups contained an aliquot of the same untreated cell samples of both experiments. Resulted peptides from each sample were labeled with an iTRAQ reagent. After labeling, peptides were mixed and subjected to strong cation exchange chromatography and separated into 30 fractions. Peptides in each fraction were desalted and concentrated using C18 spin column. Based upon the quantity, fractions with low peptide yield were pooled, which resulted into total of 12 fractions. Peptides in these fractions were further resolved on reverse phase chromatography and then analyzed on Orbitrap Velos Mass Spectrometer.

### Identification of DEPs

Mass spectra were searched against mouse protein sequences from SwissProt protein database using Mascot, X!Tandem, and Sequest search engines. Results obtained from these search engines were further combined using Scaffold 3 Q+. Total of 4336 proteins were identified and quantified with 0.5% FDR at protein level and 0.1% FDR at peptide level. Each of proteins is identified with at least 1 unique peptide having ≥95% identification probability. To allow identification of DEPs after 5 h HU210 *versus* 5 h DMSO treatment, we normalized each protein expression value of each of the two 5 h samples within each run by dividing it by the average expression value of that protein in both untreated samples of that same run.

Only proteins that were identified based on at least two different peptides were kept for further analysis. DEPs were determined *via* student's two-sided *t-*test under the assumption of equal variance. We found that the HU210-induced protein expression changes were of lower magnitude than the ones documented for the transcriptomic data. A likely explanation for this observation is that in our model system only a fraction of the total cellular proteins is directed to the *de novo* forming neurite, while a large fraction of the identified proteins would localize to the cell body. Consequently, the total changes in protein expression levels should be small and we used less stringent significant cutoffs to identify DEPs (nominal *p*-value: 0.05) and a minimum average fold change (±log_2_(1.1)). When we identified SCPs involved in NOG, we focused on the SCPs predicted from the transcriptomic changes and used the SCPs predicted from the proteomic analysis only to confirm the transcriptomic predictions. Accordingly, all except one of the genes that were selected for siRNA-based validation (see below) were predicted from the transcriptomic data analysis alone or its combination with the proteomic data analysis.

### Bioinformatics analyses: Standard and dynamic enrichment analysis

Upregulated and downregulated genes and proteins were subjected to pathway enrichment analysis using Fisher's Exact Test and the MBCO (18) (version 1.1) (https://github.com/SBCNY/Molecular-Biology-of-the-Cell) (www.mbc-ontology.org). MBCO is a strictly cell biological ontology that was designed based on standard cell biology books such as ‘Molecular Biology of the Cell’ (15) and populated by a combination of PubMed abstract text mining and statistical enrichment analysis. Since mitochondria SCPs are distributed over various MBCO branches (*i.e.*, they are children of multiple level-1 SCPs such as ‘Organelle organization’, ‘Energy generation and metabolism of cellular monomers’, and ‘Transmembrane protein import and translocation’), we generated a new level-1 mitochondria SCP (‘Structural organization of mitochondria’) and defined it to be the parent of all mitochondria related level-1 and level-2 SCPs. It was populated with all genes of the SCPs ‘Mitochondrial gene expression’, ‘Mitochondrial dynamics’, ‘Mitochondrial energy production’, ‘Mitochondrial protein import machinery’, and ‘Posttranslational protein modification in Mitochondria’. MBCO is populated with the human gene products. We used the Jackson Laboratories mouse informatics database (Mouse Genome Informatics [MGI], http://www.informatics.jax.org) and the National Center for Biotechnology Information homologene (http://www.ncbi.nlm.nih.gov/homologene/) database to replace human by their mouse orthologs. For statistical accuracy, we kept only those gene products in the MBCO that had a chance of being identified as differentially expressed (*i.e.*, only gene products whose genes are ensembl genes on the mm9 genome or only gene products whose proteins are annotated in the SwissProt protein database). Similarly, all DEGs or DEPs that are not part of the MBCO background gene product list (that contains all 13,762 gene products that were identified in at least 1 abstract during the MBCO text mining) were also removed.

Significantly upregulated or downregulated genes with a minimum log_2_ fold change of ±log_2_(1.5) were separately subjected to dynamic enrichment analysis, using MBCO level-2 and level-3 SCPs. MBCO contains predicted horizontal interactions between SCPs of the same level that can be ranked by their interaction strengths and complement the hierarchical vertical relationships between parent and child SCPs. Dynamic enrichment analysis uses these horizontal interactions to predict SCP networks that underlie whole cell function. Briefly, dynamic enrichment analysis identified those level-2 or level-3 SCPs that contain at least one input gene (*e.g.*, an upregulated gene) and combines two or three of the identified SCPs to function-specific higher-level SCPs, if they are strongly connected. To generate level-2 SCP networks, we use the top 20% of horizontal (within the same level) SCP interactions, to generate level-3 SCP networks the top 25% of interactions. Generated SCP combinations are added as new function-specific SCPs to the ontology, and the new function-specific MBCO is used for enrichment analysis of the input gene list *via* Fisher’s Exact test. Results are ranked by *p*-value and the top three level-2 or top five level-3 predictions (that are either SCPs or function-specific SCP combinations) are connected with each other, if they were predicted as part of the same function-specific SCP. See (18) for details.

Significantly, upregulated or downregulated genes with a minimum log_2_ fold change of ±log_2_(1.3) were subjected to standard enrichment analysis using MBCO and Fisher’s Exact test. All SCPs with a nominal significance of at least 5% at a particular time point were considered as significant and investigated for biological relevance.

Significantly upregulated or downregulated proteins were subjected to dynamic and standard enrichment analysis, as described above.

### siRNA testing in NOG assay

All methods used were approved by the IACUC at Icahn School of Medicine at Mount Sinai (IACUC approval# PROTO201900624). Icahn School of Medicine at Mount Sinai is AAALAC accredited facility with a full time veterinarian and veterinarian staff. All animals were maintained under humane conditions and minimized all undue suffering or pain. They were maintained in enriched environmental cages with food and water ad Librium.

Briefly, primary cortical neurons were seeded on one side of microfluidic chambers using neurobasal media supplemented with B27, L-660 glutamine, and penicillin-streptomycin (NB+++). The two sides of the chambers are separated by a 150 μm microgroove sidewall that contains slits that are too small for whole cells to pass but large enough to allow neurite growth into the other side of the chamber. Consequently, neurites will grow across the neurite side, allowing an easy quantification of outgrowth length. About 1.5 h to 2 h after plating, we transfected the primary neurons with a pool of four different siRNAs (Dharmacon AccellsiRNA delivery SMART Pools). In total, we used two different siRNA batches for our experiments. The next day transfection media was exchanged for NB+++ media that contained 25% astrocyte-conditioned media. To generate astrocyte-conditioned NB+++, we incubated primary astrocytes for at least 12 h with NB+++, followed by sterile filtration of the used NB+++. Astrocyte-conditioned media contained astrocyte-secreted metabolites and growth factors that support survival of primary cortical neurons. To allow the gene silencing effect to get established, we continued to incubate the cells until 48 h after siRNA transfection. Basal outgrowth not attributable to the siRNAs was removed by axotomy afterward. Another 48 h later (total of 96 h after transfection), we stopped the experiments by exchanging the media for PBS containing 4% paraformaldehyde and 4% sucrose. Cells and neurites were stained with anti-βIII tubulin (Tuj1; Covance). To quantify NOG, we imaged the slides on a LSM880 confocal microscope (10× or 20×, zoom ins: 31× or 63×). For details, see (47). Images were obtained with the cell body side of the chamber on the left and the neurite side on the right side of the image.

### Analysis of NOG

Before analysis of NOG effects of the different siRNAs, we submitted all images (generated from 246 biological replicates) to a computer-assisted quality control step. In a small number of samples, the axotomy damaged the cell body site, so that the NOG results were not interpretable. An axotomy that damages the cell body site removes cell bodies from the area right next to the microgroove wall. Consequently, tubulin fluorescence intensity is less at this area and the intense line of tubulin fluorescence is retracted from the microgroove. Based on these criteria, we generated a computational pipeline to automatically identify samples with damaged cell bodies. We used Image J software (https://imagej.nih.gov/ij/) and the Plot Profile function to analyze the cell body site, starting at the left edge of the microgroove. This function calculates the average intensity of all pixels at a certain distance from the microgroove. All distances were converted to 0.1 μm steps, assuming a linear increase or decrease between any two measured adjacent intensity values. In case of multiple images of one microfluidity chamber, we calculated the average intensity for each image (without double quantification of overlapping areas in the images) and generated the average intensities for the whole chamber based on the intensities of the individual images. The intention of our algorithm was to identify those samples where the intense line of tubulin labeling was too far away from the microgroove. For this, we focused on an area of up to 100 μm left of the microgroove, that is, of an area that is 2/3 as wide as the microgroove itself. Average cell body intensities were normalized toward the highest intensity within this area of each sample (Fig. S3*A*). We screened for intensity peaks within this area and determined the highest peak within the first 50 μm left of the microgroove. A peak was defined as the highest point in the middle of a 5 μm interval. If the normalized intensity of this peak was not at least 85%, our algorithm concluded that the intense line of tubulin staining is too far away from the microgroove wall and the cell body site of this sample was labeled damaged. Computational proposals were manually confirmed or—in a few cases—corrected (Fig. S3*B*). In total, we removed 39 from the initial 246 biological replicates during this quality control step.

Manual investigation also identified 22 additional replicates that were removed for reasons other than a cell body site that was damaged during axotomy, for example, low or no growth in the whole replicate (Fig. S3*C*).

Finally, we had to remove 24 and 13 high quality biological replicates because the corresponding controls of the same experiment were removed or only one biological replicate was left over for a tested gene, respectively (Fig. S3*C*).

Since our validation experiments were based on two different siRNA batches, we documented how many replicates of each gene were generated from each of those batches (Table S7). In the case of 16 genes, the replicates that were subjected to statistical analysis after quality control were generated from both batches.

To quantify the effect of gene knockdown on NOG, we quantified the average fluorescence intensity at distances to the right of the microgroove wall. As described for the cell body sites, we converted all distances to distances separated by 0.1 μm (starting with 0 μm) and merged the results of multiple images of the same sample. Each experiment contained one to three controls. In case of multiple controls, we calculated the mean average intensities that were used for all further analyses. To allow comparison of the results across different experiments, we normalized all average intensities of each siRNA or control sample of the same experiment toward control average expression at 100 μm. Quantified NOG results showed interexperimental variation. To compare the results across different experiments, we identified those distances from the microgroove at which the control intensity had fallen to 75%, 50%, and 25%. The intensities at these distances of each siRNA-treated sample were identified and compared for each gene across the different experiments. Outliers at each distance were identified for genes with at least four samples using Dixon’s Q-test (confidence interval, 0.9) and removed from further analysis (Fig. S3*D*). One sample two-sided *t* test (equal variance) was used to determine whether the sample outgrowth was significantly different from 75%, 50%, or 25%. Any gene knockdown with a nominal *p*-value equal to or less than 0.05 for at least one distance was considered as significantly influencing NOG. Similarly, any gene knockdown with a nominal *p*-value larger than 0.05 but smaller or equal to 0.1 was considered to possibly influence NOG.

In summary, we subjected 11, 10, 11, and 4 genes with 2, 3, 4, and 5 biological replicates, respectively, to the statistical analysis described previously. Only 2 of the 11 genes where only 2 replicates survived the morphological quality control were significant and one of these is supported by prior experimental data from other labs (66). Hence, using multiple conservative selection criteria a total 27 of 36 tested genes showed effects on NOG that we predicted from our prior analysis (Table S8).

### Dynamical modeling

Details of the modeling approach used for modeling outgrowth have been published in Yadaw *et al* (47). Briefly, our multicompartment ordinary differential equation model simulates the interaction of membrane lipid and protein synthesis, vesicle transport, and microtubule growth during NOG (Fig. 4*A*). Newly synthesized membrane and transmembrane proteins are added to the TGN. Vesicles that bud from the TGN incorporate the membrane lipids and proteins. The budding vesicles contain the transmembrane kinesin receptor that binds to kinesin, thereby allowing anterograde movement along the microtubule scaffold of the neurite toward the growth cone at the neurite tip. Within the growth cone, the vesicles dissociate from the microtubule and fuse with the plasma membrane by the interaction of vesicle SNAREs with membrane SNAREs. Any lipid membrane excess is transferred to the neurite causing its growth. Retrograde vesicles bud from the growth cone membrane, travel back to the cell body *via* dynein-mediated vesicle transport, and fuse with the TGN *via* a different set of SNAREs. To allow a systematic analysis of the underlying dynamics, we developed an analytical solution that predicts numerical outcomes of our dynamical model with high accuracy. The analytical solution allows analysis of parameter constellations that allow NOG at any given velocity and in agreement with additional model constraints that we curated from the literature. For example, one of the constraints demands that only 10% of all anterograde vesicles in the neurite are bound to the microtubule and actively move forward, while the other 90% form a vesicle reservoir in the growing neurite. Consequently, the growing neurite acts as a sink for vesicle membrane and proteins, so that membrane lipid and protein synthesis needs to be adjusted accordingly. The analytical solution also allows identification of parameter constellations that lead to stalling of the numerical simulation and diminished neurite growth, indicated by parameters with negative values or values that exceed predefined sets of maximum values. In summary, our top-down approach does not predict the outcome of different parameter constellations on a cellular phenotype but predicts the parameter constellations that allow generation of a predefined cellular phenotype. For a flow chart of our top-down approach, see Figure 2 of Yadaw *et al* (47).

Here, we used the analytical solution to investigate the importance of vesicle back transport from the growth cone to the TGN for NOG. We analyzed the effect of different vesicle back transport rates on the total number of needed vesicle SNAREs and kinesin receptors, in dependence of the anticipated NOG velocity. Methodologically, we defined the anticipated outgrowth velocities and the back transport rates. For each combination, our analytical solution predicts the amount of vesicle SNAREs and kinesin receptors that allow outgrowth under these conditions. Vesicle SNAREs and kinesin receptors continuously cycle between the TGN and the growth cone membrane. Consequently, these molecules can be localized at the TGN, at anterograde or retrograde vesicles and the growth cone membrane. We here document the total number of those molecules, independently of their localization. Since the number of vesicle SNAREs and kinesin receptors continuously increase during the simulation (due to the increasing number of vesicles that reside in the growing neurite), we focused on the total amount of vesicle SNAREs and kinesin receptors at the beginning of the simulation. We also described minimum back transport rates for each analyzed NOG velocity. Below these minimum back transport rates, the predicted vesicle SNAREs and kinesin receptors exceeded the predefined maximum number, indicating stalling of NOG.

## Data availability

RNA-seq data is available at NCBI GEO under the accession GSE198622. The mass spectrometry proteomics data have been deposited to the ProteomeXchange Consortium via the PRIDE partner repository with the dataset identifier PXD036706 (10.6019/PXD036706). Microscopic images and image quantifications are available upon request (Please contact the corresponding author.).

## Code availability

MATLAB code of the initial and extended dynamic model is available at Github (https://github.com/SBCNY/Dynamical-Model-of-Neurite-Outgrowth and https://github.com/SBCNY/Extended_dynamical_model_of_neurite_outgrowth, respectively).

## Supporting information

This article contains supporting information (67, 68, 69, 70, 71, 72, 73, 74, 75, 76, 77, 78, 79, 80, 81, 82, 83, 84, 85, 86).

## Conflict of interest

The authors declare that they have no conflicts of interest with the contents of this article.
