# Supplementary material for: Whole cell response to receptor stimulation involves many deep and distributed subcellular biochemical processes
Source: J Biol Chem. 2022 Aug 1;298(10):102325. doi: 10.1016/j.jbc.2022.102325 (PMC9520007; doi:10.1016/j.jbc.2022.102325)
Supplement: Supplemental Figures S1–S4 [file mmc1.pdf]

Supplemental information for

Whole cell response to receptor stimulation involves many deep and distributed subcellular biochemical processes

Running Title: Integration of subcellular process for cell level responses

Key Words: subcellular pathways, transcriptomics, bioinformatics, dynamical modeling, neurite outgrowth

Jens Hansen[1], Mustafa M. Siddiq[1], Arjun Singh Yadaw[1], Rosa E. Tolentino[1], Vera Rabinovich[1], Gomathi Jayaraman[1], Mohit Raja Jain[2,3], Tong Liu[2], Hong Li[2],Yuguang Xiong[1], Joseph Goldfarb[1], Ravi Iyengar[1]*

[1]Department of Pharmacological Sciences and Institute for Systems Biomedicine, Icahn School of Medicine at Mount Sinai, New York, NY 10029, United States.

[2]Department of Microbiology, Biochemistry and Molecular Genetics, Rutgers University, New Jersey Medical School, Newark, NY, 07103, United States.

[3]Current address: Thermo Fisher Scientific, 1400 North Point Parkway, Suite 10, West Palm Beach, Fl 33407, United States.

*To whom correspondence should be addressed: Ravi Iyengar, Department of Pharmacological Sciences, 1425 Madison, Rm 12-70, Icahn School of Medicine at Mount Sinai, New York NY 10029, United States, Voice: 212-659-1707, E-mail: ravi.iyengar@mssm.edu

Further descriptive analyses of SCPs identified by siRNA knockdown

Regulation of mRNAs

In parallel to the up-regulation of ribosomal genes after 4h HU210 treatment, we observed multiple downregulated genes that participate in bulk mRNA degradation (Suppl. Figure 4A). Our results suggest that an increase in gene expression needed for continuous synthesis of neurite components for the following neurite growth phase (6h) is facilitated by an increase in ribosomal translation and mRNA lifetime. Down-regulated genes after 4h HU210 treatment are part of multiple SCPs that participate in mRNA degradation at multiple consecutive steps (67): initial mRNA-polyA tail trimming, bulk mRNA polydeadenylation by the CCR4-NOT complex, mRNA decapping and exosome-independent degradation of deadenylated and polyuridylated mRNA (68). The CCR4-NOT complex regulates numerous steps during mRNA biogenesis including transcription, mRNA export and mRNA degradation (22,69-71). Three of the five down-regulated CCR4-NOT complex subunits have 3'-5' poly(A) exoribonuclease activity (Cnot6, Cnot6L, Cnot7) (70), suggesting that down-regulation of these complex components increases mRNA lifetime.
siRNA knock down of the putative catalytic component Dis3 of the exosome complex (72,73) that is involved in another degradation pathway of polydeadenylated mRNA decreases NOG (Figure 3B, Suppl. Figure 3D), indicating that mRNA degradation, possibly of mRNAs that contain errors, is still a necessary function to secure neurite outgrowth.

Mitochondrial Functions

DEGs obtained after 4h HU210 treatment that are involved in structural mitochondrial organization suggest a shift from Rhot1 to Rhot2 mediated anterograde mitochondrial transport, a shift from mitochondrial fission to mitochondrial fusion, the down-regulation of mitochondrial gene expression, and activity changes in SCPs that are involved in mitochondrial protein import, mitochondrial gene expression and oxidative phosphorylation (Suppl. Figure 4B). Knock down of three mitochondrial genes (Rhot1, Tufm and Mtx1) significantly inhibited NOG, while knock down of another gene (Mfn2) may inhibit NOG (based on a p-value close to significance) (Figure 3B, Suppl. Figure 3D), documenting the importance of the SCPs mitochondrial anterograde transport, fusion, translation and protein import. Mitochondria populate the growing neurite where they increase their length through fusion (74). Our results suggest that mitochondria are first prepared for populating the neurite before the neurite enters its major growth phase, since mitochondrial genes are up-regulated before the up-regulation of genes involved in membrane synthesis and vesicle trafficking (Figures 2D/E/F). This interpretation complements morphological observations showing mitochondria, peroxisomes and Golgi vesicles accumulation at the base of that neurite that becomes the future axon before it enters its major growth phase (28,29).

Pyrimidine Biosynthesis Pathways

Pyrimidine synthesis and salvage was predicted to be up-regulated after 4h HU210 treatment. Pyrimidines are substrates for multiple sub-cellular pathways such as DNA and RNA synthesis and are co-factors during the synthesis of phosphatidylethanolamine (PE), phosphatidylcholine (PC) and complex gangliosides (Suppl. Figure 4C) (30). The up-regulation of pyrimidine synthesis and salvage could therefore support increased gene expression by delivering the building blocks for mRNA synthesis. Additionally, an increased pool of the pyrimidine nucleoside triphosphates CTP and UTP could support membrane lipid and ganglioside synthesis, SCPs that are up-regulated after 6h HU210 treatment (Suppl. Figure 4D). CTP is a co-factor for neuronal phosphatidylethanolamine and -choline synthesis via the Kennedy pathway (37) and uridine induces intracellular CTP increase and neurite outgrowth (75). UTP and CTP are co-factors for ganglioside synthesis (76). Though pyrimidine de novo synthesis was also up-regulated, we could only document the dependence of NOG on pyrimidine salvage, but not on pyrimidine de-novo synthesis (Figure 3B, Suppl. Figure 3D). Knock down of thymidine phosphorylase (Tymp), an enzyme of the salvage pathway, decreased NOG. In contrast, knock down of the enzyme carbamoyl-phosphate sythetase 2, aspartate transcarbamylase, and dihydroorotase (Cad) that is involved in pyrimidine de-novo synthesis showed no effect. Knock down of dihydroorotate dehydrogenase (Dhodh), the second tested enzyme of the de-novo synthesis pathway, even increased NOG. These results are in agreement with a mechanism that favors pyrimidine recycling. De novo synthesis of pyrimidines is associated with high energy cost, so pyrimidine recycling is favored to maintain a sufficient pool for pyrimidine dependent pathways (37). The source for recycled substrates might either be pyrimidine metabolites that were generated in the same cell (e.g. after DNA/RNA degradation) or metabolites that were produced by astrocytes, since we used astrocyte conditioned media, to generate an environment that resembles the in-vivo situation in the CNS.

Lipid Biosynthesis Pathways

The distribution of up- and down-regulated genes within membrane lipid synthesis pathways suggests an increase in the synthesis of C18 ceramide and complex gangliosides (Suppl. Figure 4D) and a shift in phospholipid synthesis from phosphatidylcholine (PC) to phosphatidylethanolamine (PE) and phosphatidylserine (PS). Genes involved in lipid synthesis pathways are up-regulated along with genes involved in vesicle transport, suggesting the functional coupling of pathways for membrane synthesis and delivery of these membranes to the growth cone.

Complex gangliosides contribute 10 to 20% of lipids in neuronal membranes (34) and are synthesized using ceramide as a substrate (77). They are primarily localized in the outer leaflet of the plasma membrane (35), suggesting that ceramide and ganglioside synthesis are up-regulated to generate the building blocks of the outer leaflet of the growing neurite. In agreement with these observations, we find that knock down of genes that are involved in ganglioside synthesis, either

as an enzyme (Ugcg) (78) or as a lipid transport protein (Plekha8) that directs glycosylceramide from the cis-Golgi to the synthesis site of complex gangliosides at the trans-Golgi (40-42), inhibit neurite outgrowth (Figure 3B, Suppl. Figure 3D). In contrast, the knock down of Col4a3bp, a lipid transport protein that directs glycosylceramide to sphingomyelin synthesis sites at the Golgi (41,79,80) did not influence NOG (Figure 3B, Suppl. Figure 3D). Our results are in agreement with experimental data suggesting neuronal sphingomyelin synthesis at a non-Golgi location (81,82).

PE and PS mainly localize to the inner leaflet of the plasma membrane (83), suggesting that the up-regulation of enzymes involved in PE and PS synthesis via the Kennedy pathway increases the production of the building blocks of the inner leaflet of the growing neurite. PC, the most abundant phospholipid in cell membranes, is largely localized at the ER (84) and serves as a storage reservoir for choline that is used for synthesis of the neurotransmitter acetylcholine (85,86). The two different arms of the Kennedy pathway that synthesize PE (that can be converted into PS) and PC both utilize diacylglycerol (DAG) as a substrate and the pyrimidine nucleoside triphosphate CTP as a co-factor (37). The predicted down-regulation of PC synthesis might further favor the production of PE and PS by increasing the availability of both molecules for PE synthesis. Knock down of Pcyt2, an enzyme involved in PE synthesis via the Kennedy pathway, may decrease NOG, since we could document reductions of NOG in three independent experiments, though the p-value was above our two significant cutoffs (0.125) due to large experimental variations (Figure 3B, Suppl. Figure 3D). Similarly, the knock down of Pcyt1a, an enzyme involved in PC synthesis via the Kennedy pathway, may also decrease NOG (p-value: 0.069). Although our DEGs suggest a shift from PC to PE and PS synthesis to increase neurite membrane production, NOG may still depend on a sufficient supply of PC as the most abundant phospholipid in cell membranes (60).

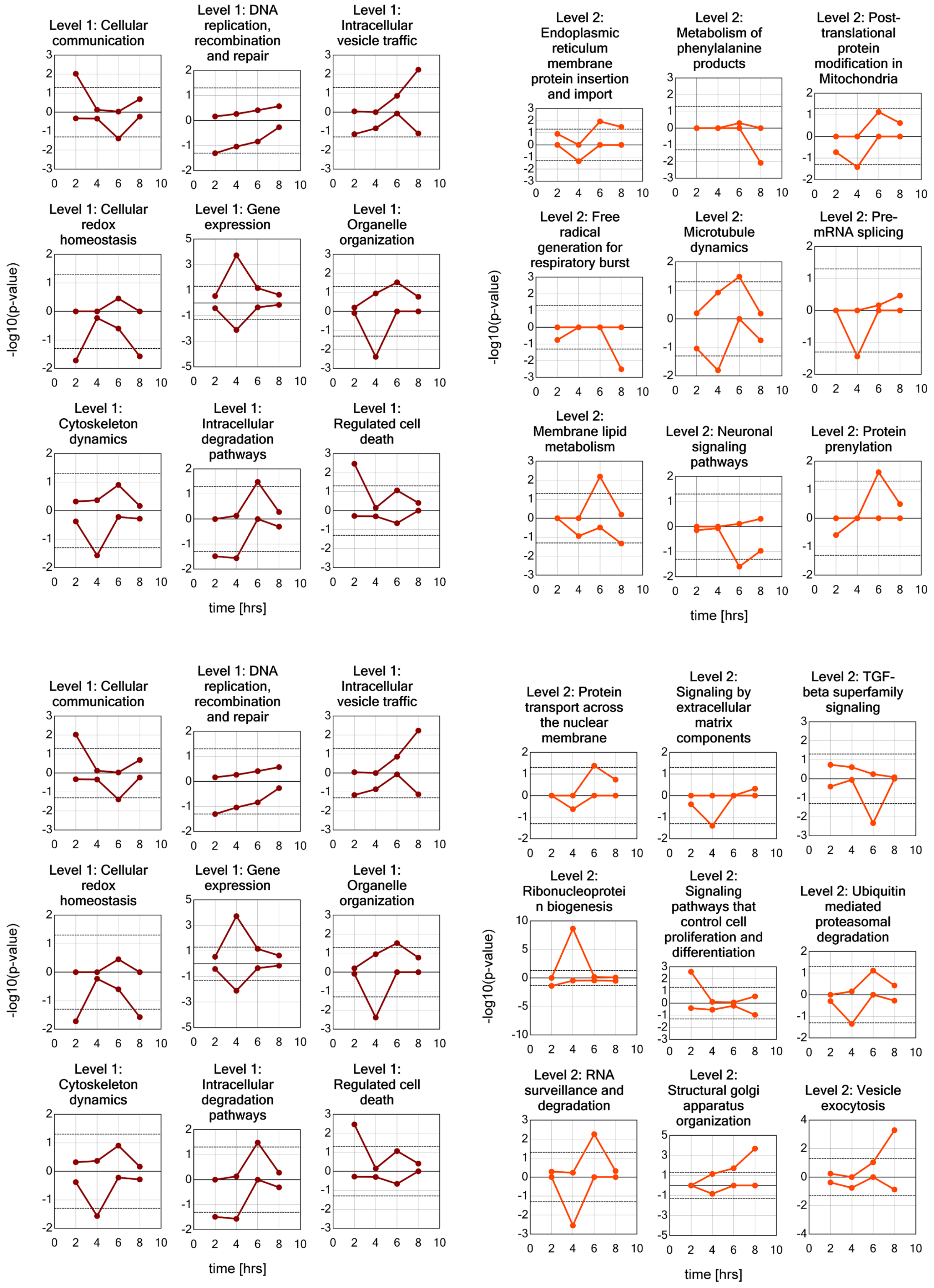

Suppl. Figure 1

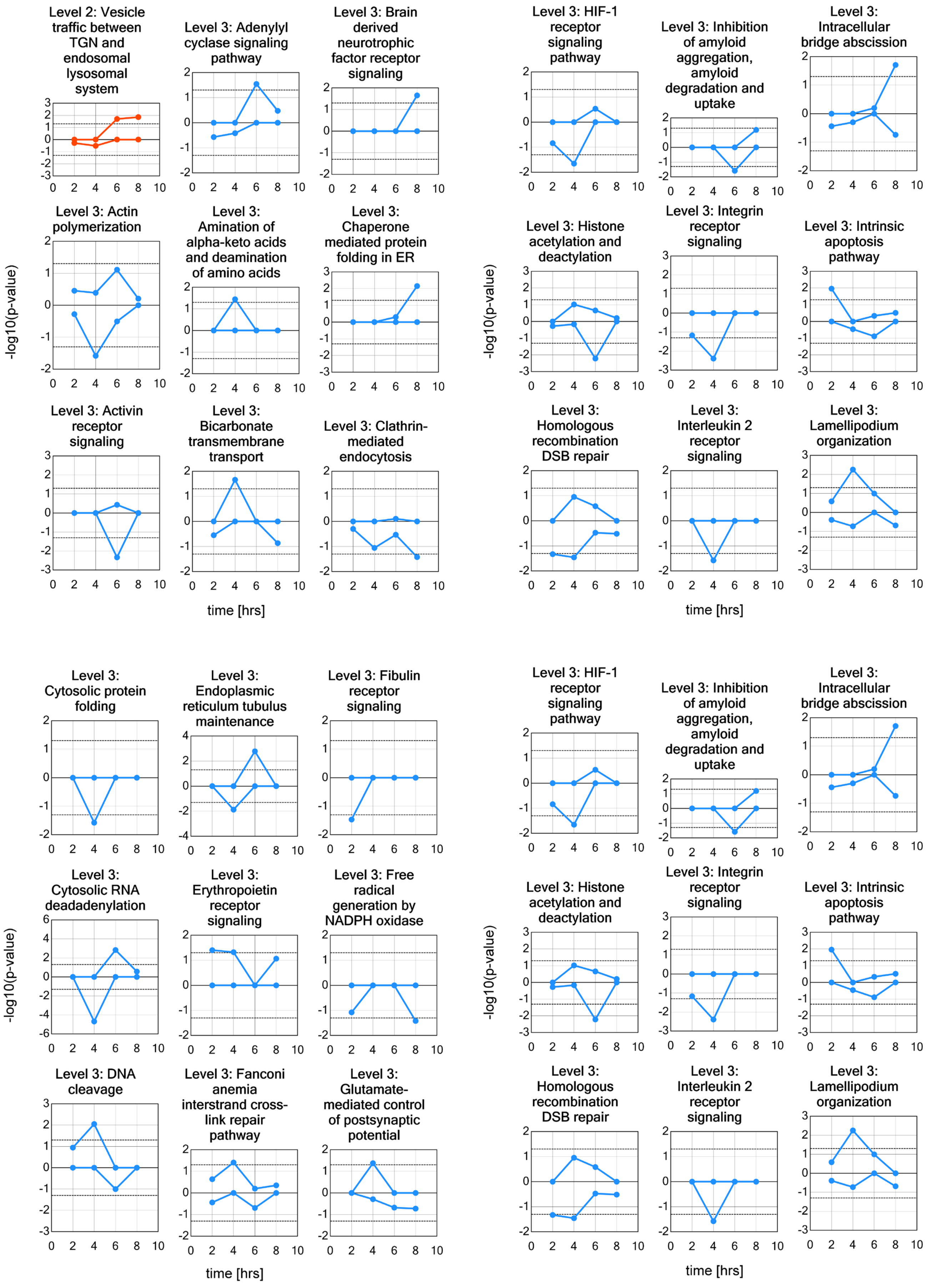

Suppl. Figure 1

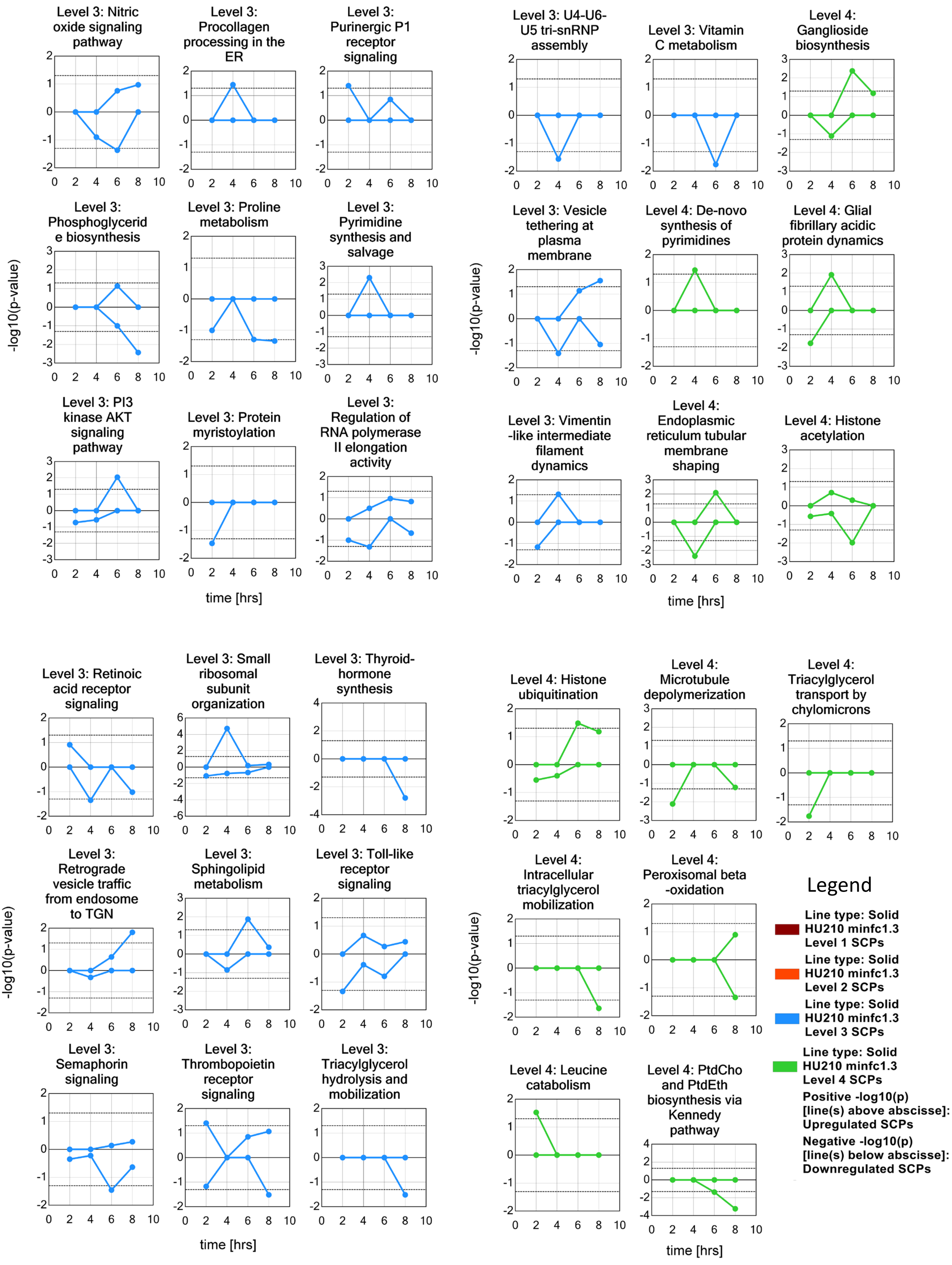

Suppl. Figure 1

Suppl. figure 1: Time courses of all SCPs predicted based on standard enrichment analysis.
For details see figure 2E.

A

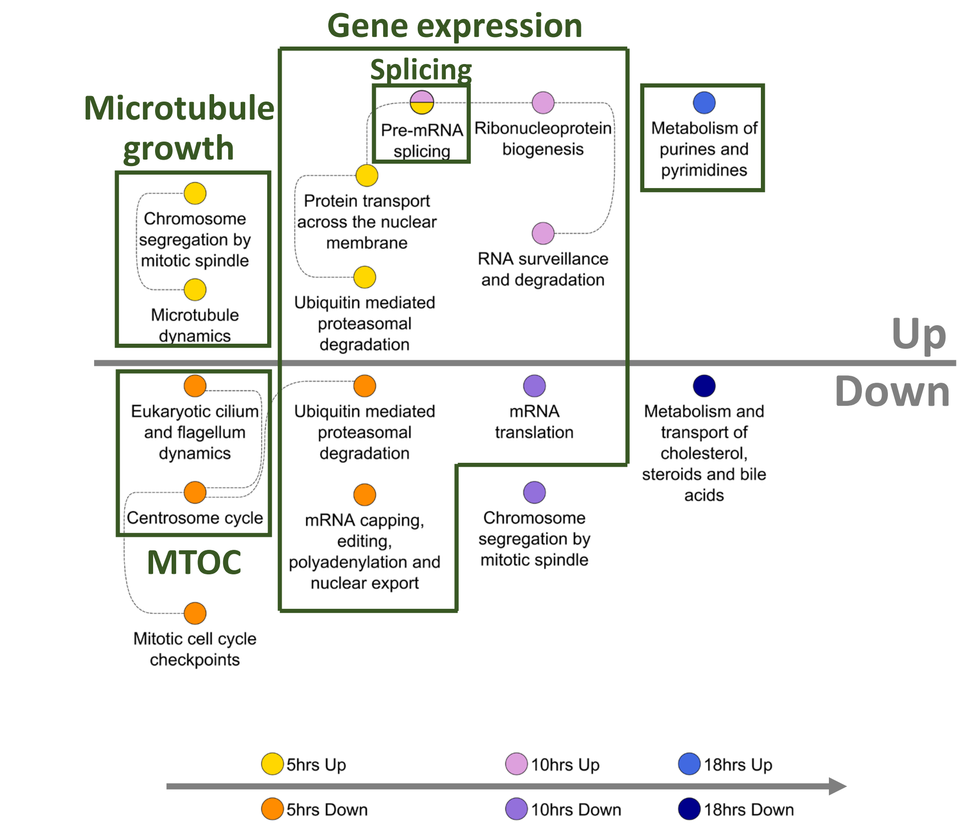

B

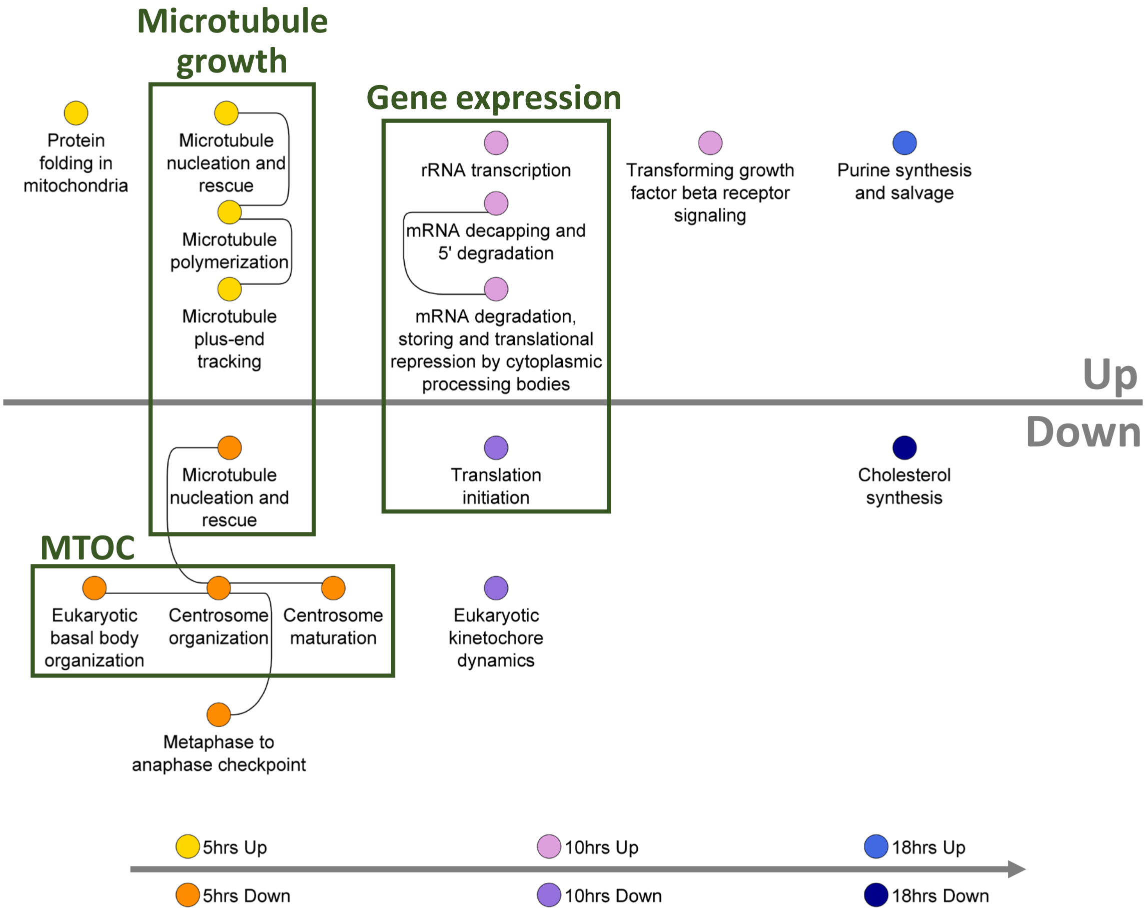

Suppl. Figure 2

C

HU210 proteomics - 5 hrs - Up

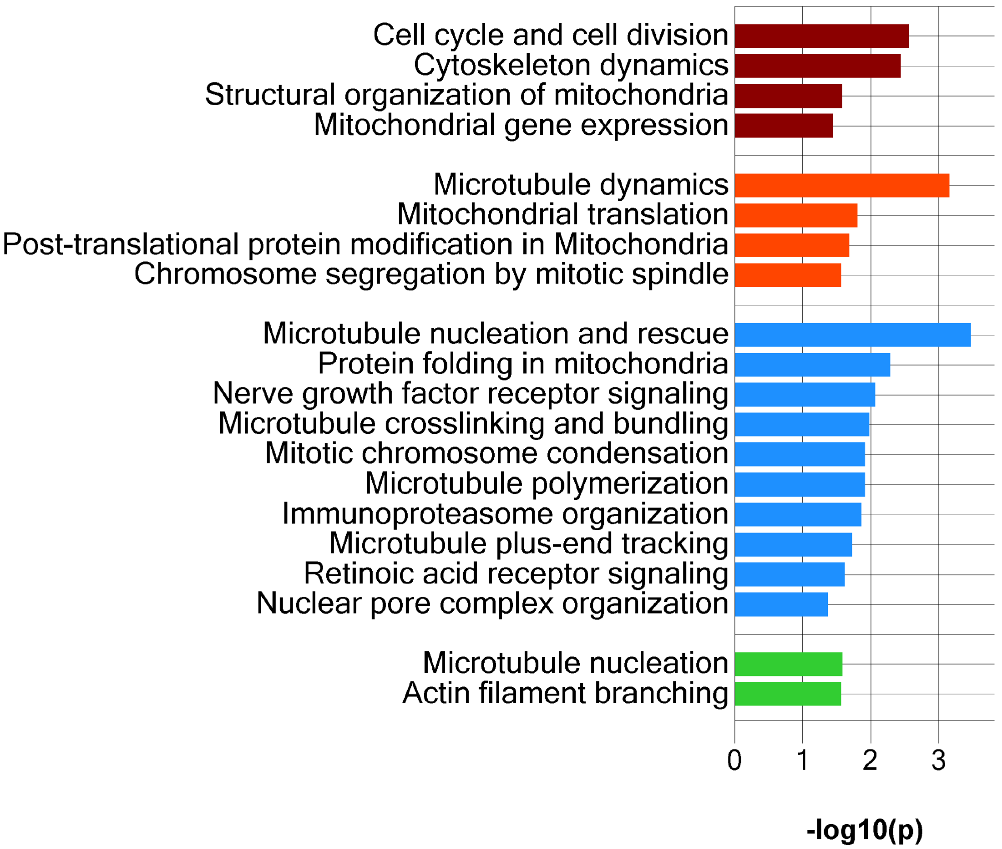

HU210 proteomics - 5 hrs - Down

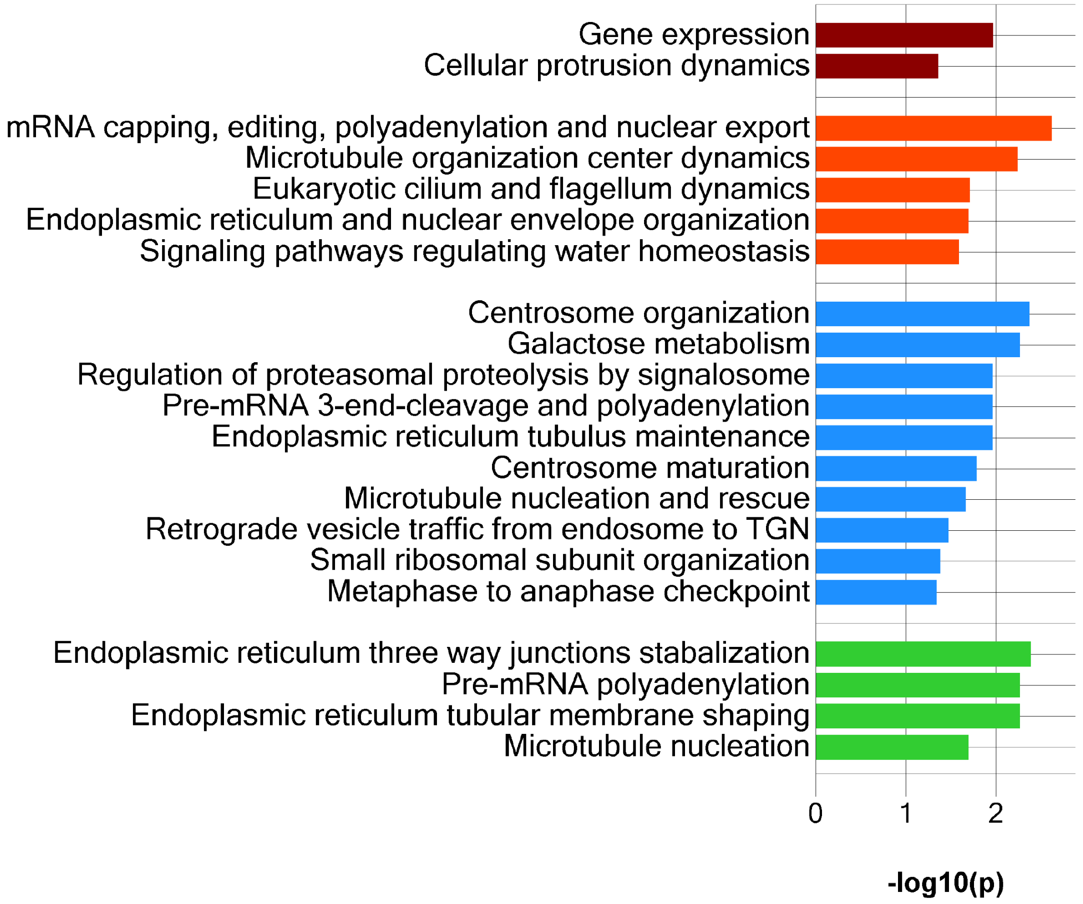

Suppl. Figure 2

D

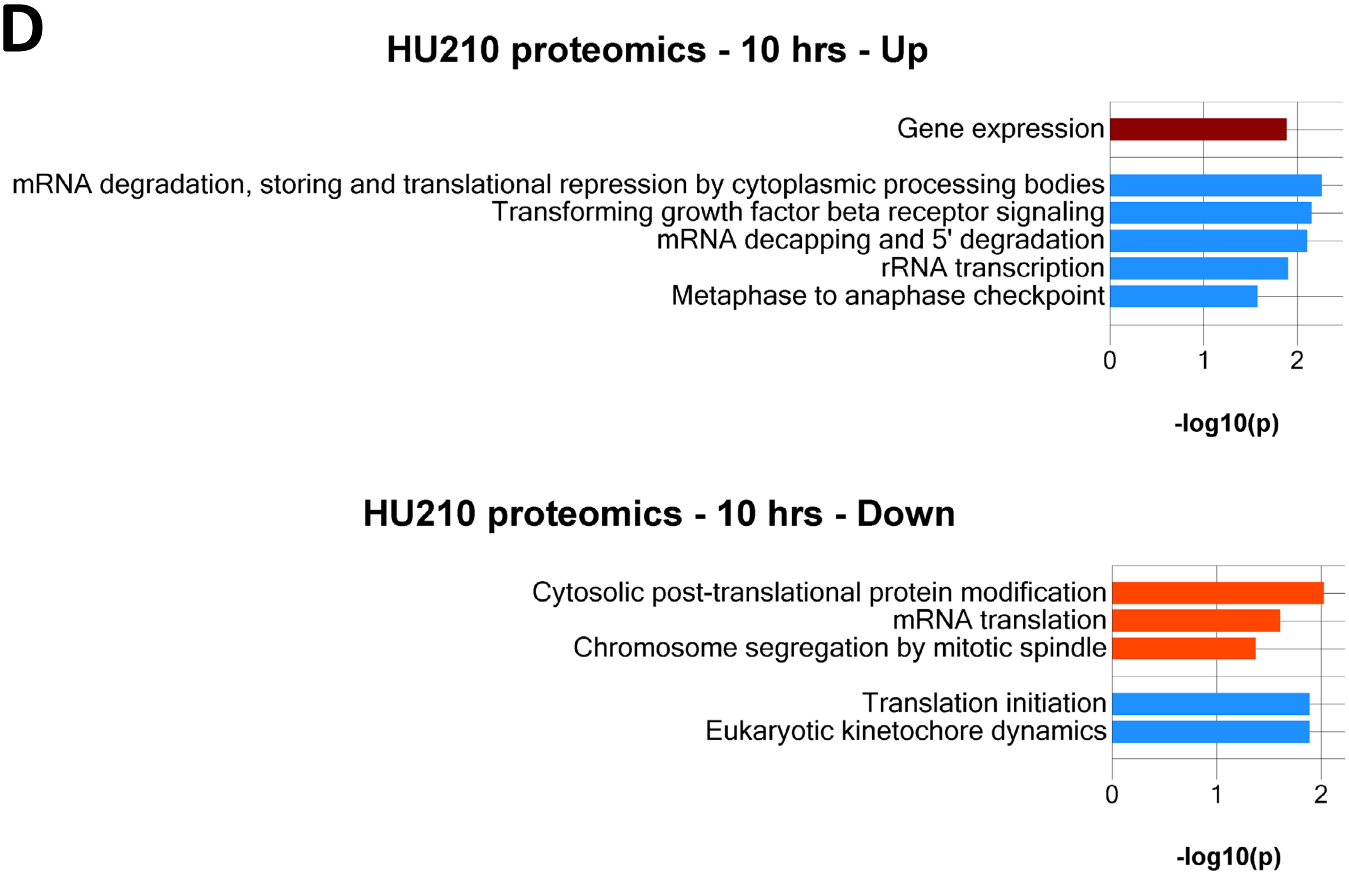

E

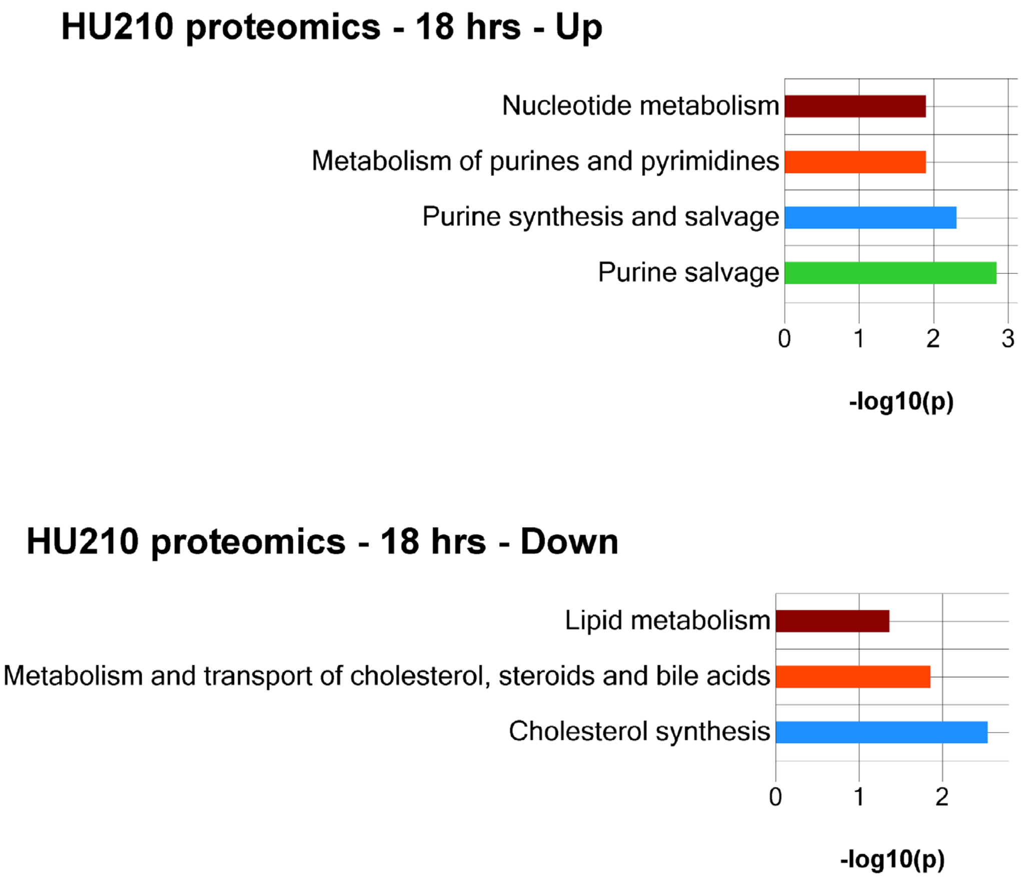

Suppl. Figure 2

Suppl. figure 2: Enrichment analysis of differentially expressed proteins identified by discovery proteomics. Up- and downregulated proteins of each timepoint were subjected to dynamic enrichment analysis using MBCO **(A)** level-2 and **(B)** level-3 SCPs. Bardiagramm show –log10 P-values of the top level-1 (dark red), -2 (red), -3 (blue) and -4 (green) SCPs identified by standard enrichment analyses of up and down regulated DEPs after 5h **(C)**, 12h **(D)** and 18h **(E)** HU210 treatment.

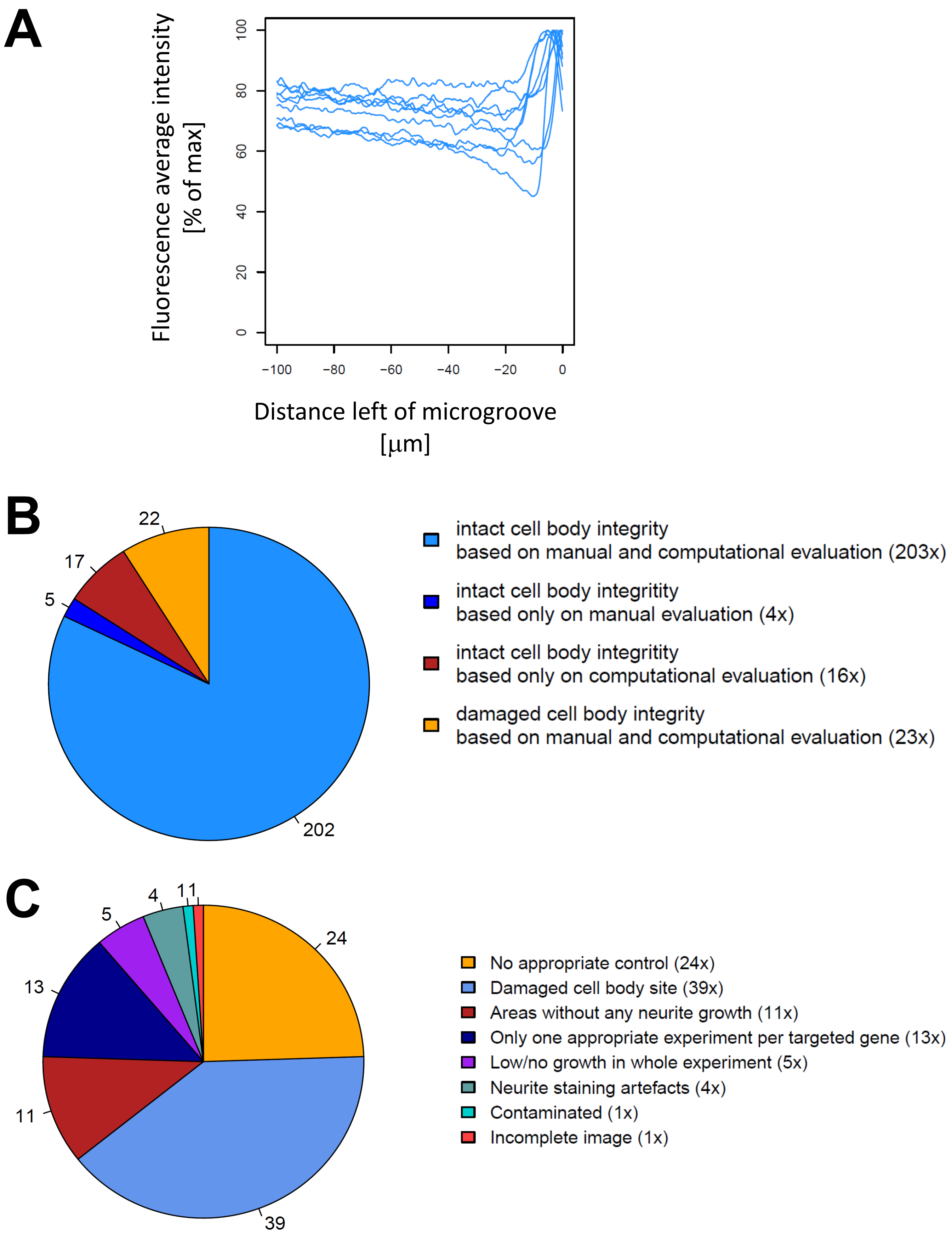

Suppl. Figure 3

D

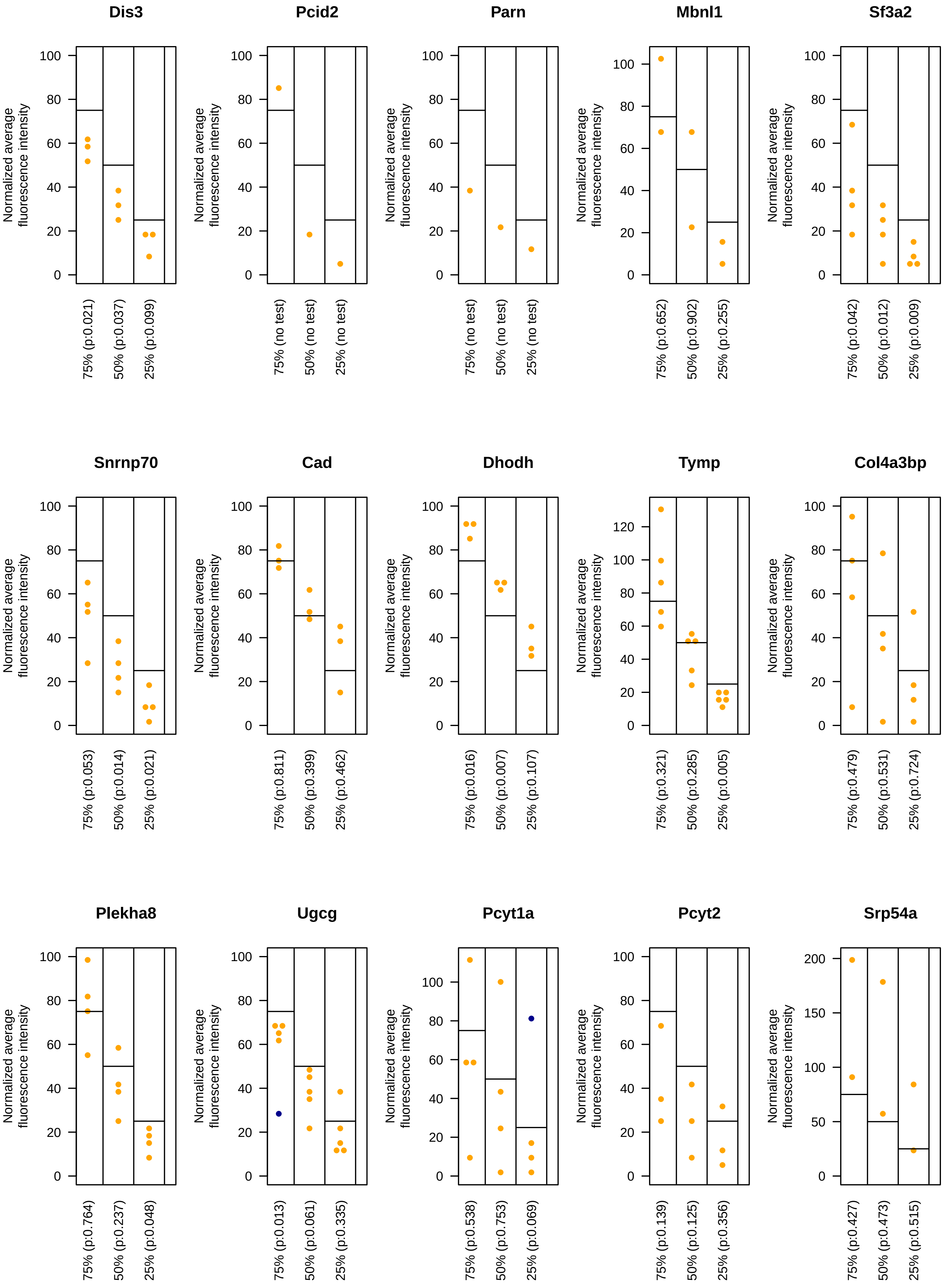
Dis3
Normalized average fluorescence intensity
75% (p:0.021)
50% (p:0.037)
25% (p:0.099)
Pcid2
75% (no test)
50% (no test)
25% (no test)
Parn
75% (no test)
50% (no test)
25% (no test)
Mbnl1
75% (p:0.652)
50% (p:0.902)
25% (p:0.255)
Sf3a2
75% (p:0.042)
50% (p:0.012)
25% (p:0.009)
Snrnp70
75% (p:0.053)
50% (p:0.014)
25% (p:0.021)
Cad
75% (p:0.811)
50% (p:0.399)
25% (p:0.462)
Dhodh
75% (p:0.016)
50% (p:0.007)
25% (p:0.107)
Tymp
75% (p:0.321)
50% (p:0.285)
25% (p:0.005)
Col4a3bp
75% (p:0.479)
50% (p:0.531)
25% (p:0.724)
Plekha8
75% (p:0.764)
50% (p:0.237)
25% (p:0.048)
Ugcg
75% (p:0.013)
50% (p:0.061)
25% (p:0.335)
Pcyt1a
75% (p:0.538)
50% (p:0.753)
25% (p:0.069)
Pcyt2
75% (p:0.139)
50% (p:0.125)
25% (p:0.356)
Srp54a
75% (p:0.427)
50% (p:0.473)
25% (p:0.515)

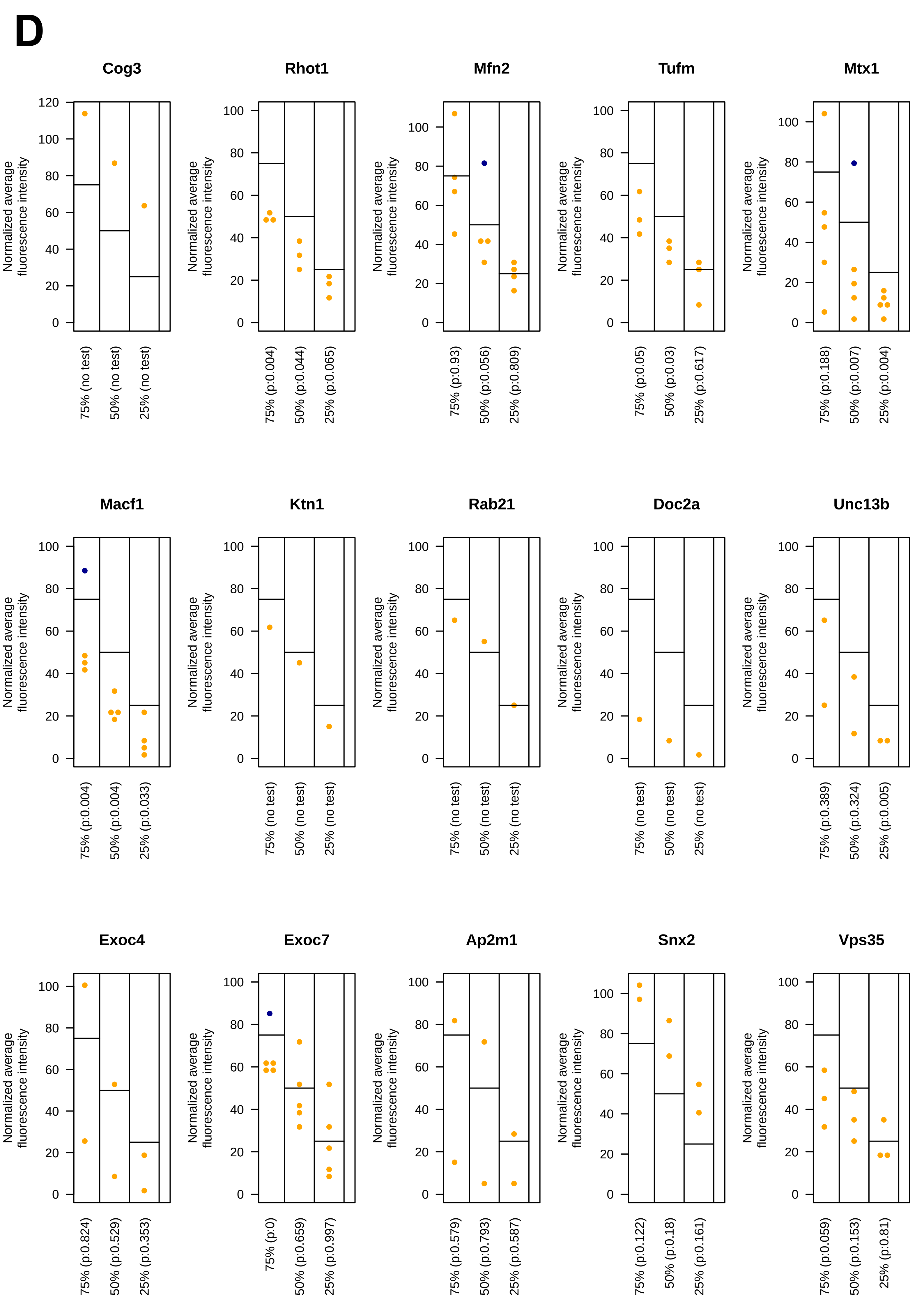

D
Cog3
Rhot1
Mfn2
Tufm
Mtx1
Macf1
Ktn1
Rab21
Doc2a
Unc13b
Exoc4
Exoc7
Ap2m1
Snx2
Vps35
Normalized average fluorescence intensity
75% (no test)
50% (no test)
25% (no test)
75% (p:0.004)
50% (p:0.044)
25% (p:0.065)
75% (p:0.93)
50% (p:0.056)
25% (p:0.809)
75% (p:0.05)
50% (p:0.03)
25% (p:0.617)
75% (p:0.188)
50% (p:0.007)
25% (p:0.004)
75% (p:0.004)
50% (p:0.004)
25% (p:0.033)
75% (p:0.389)
50% (p:0.324)
25% (p:0.005)
75% (p:0.824)
50% (p:0.529)
25% (p:0.353)
75% (p:0)
50% (p:0.659)
25% (p:0.997)
75% (p:0.579)
50% (p:0.793)
25% (p:0.587)
75% (p:0.122)
50% (p:0.18)
25% (p:0.161)
75% (p:0.059)
50% (p:0.153)
25% (p:0.81)

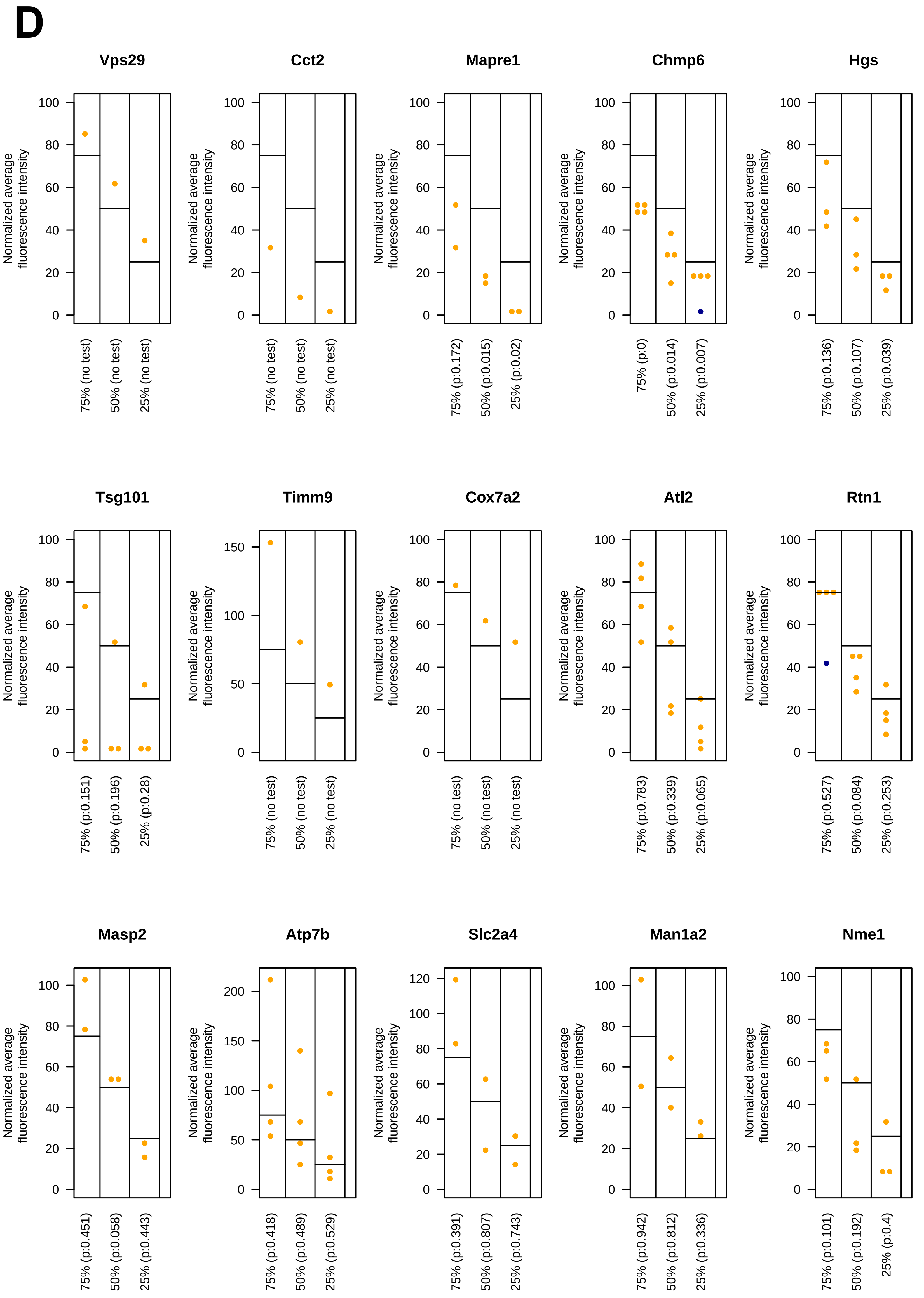
D
Vps29
Cct2
Mapre1
Chmp6
Hgs
Normalized average fluorescence intensity
75% (no test)
50% (no test)
25% (no test)
75% (p:0.172)
50% (p:0.015)
25% (p:0.02)
75% (p:0)
50% (p:0.014)
25% (p:0.007)
75% (p:0.136)
50% (p:0.107)
25% (p:0.039)
Tsg101
Timm9
Cox7a2
Atl2
Rtn1
75% (p:0.151)
50% (p:0.196)
25% (p:0.28)
75% (p:0.783)
50% (p:0.339)
25% (p:0.065)
75% (p:0.527)
50% (p:0.084)
25% (p:0.253)
Masp2
Atp7b
Slc2a4
Man1a2
Nme1
75% (p:0.451)
50% (p:0.058)
25% (p:0.443)
75% (p:0.418)
50% (p:0.489)
25% (p:0.529)
75% (p:0.391)
50% (p:0.807)
25% (p:0.743)
75% (p:0.942)
50% (p:0.812)
25% (p:0.336)
75% (p:0.101)
50% (p:0.192)
25% (p:0.4)

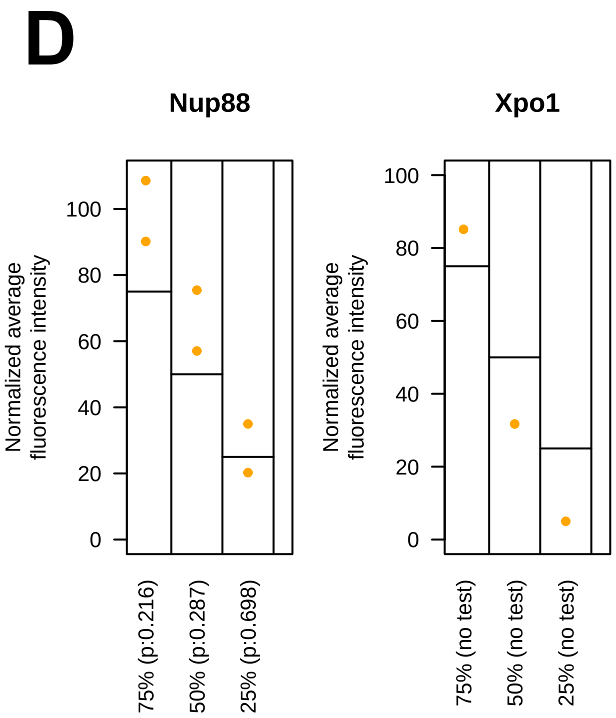

D
Nup88
Xpo1
Normalized average fluorescence intensity
0
20
40
60
80
100
75% (p:0.216)
50% (p:0.287)
25% (p:0.698)
75% (no test)
50% (no test)
25% (no test)

Suppl. figure 3: Quality control and analysis of neurite outgrowth assays. (A) During plating cells will occupy every space available within the cell body site of the chamber including those right in front of the microgroove. Microscopic analysis will document uniform tubulin staining and an intense line of tubulin staining right in front of the microgroove wall (see Figure 3A). In a few cases the cell body site was damaged during the axotomy, resulting in a removal of cells in front of the microgroove. Consequently, the staining will be less uniform and the intense line of tubulin staining will appear at an increased distance from the microgroove wall. To identify samples where the cell body side has been damaged during axotomy, we quantified fluorescence intensities on the cell body site and calculated the average intensity at each distance up to 100 μm left of the microgroove wall. All average intensities for one outgrowth chamber were normalized towards the highest intensity. Intact cell body sites show a fluorescence intensity peak right in front of the microgroove wall as shown for one typical experiment (each blue line represents one targeted or scramble siRNA treated sample). This peak corresponds to the intense line of tubulin staining. If the cell body site was damaged during axotomy, this peak will be farer away from the microgroove wall. Our algorithm was searching for the highest peak within the first 50 μm left to the microgroove wall (i.e. within a distance that corresponds to 1/3 of the microgroove wall width of 150 μm). If the normalized intensity of that peak was at least 85%, our algorithm labeled the cell body site as intact and as non-intact otherwise. **(B)** Computational predictions of cell body integrities were manually verified. In 20 out of 246 manually assigned cell body integrity differed from the computationally predicted ones. In these cases we continued with the manual quantifications. **(C)** Any samples with a damaged cell body site as well as any experiments that did not contain controls with intact cell body sites were removed from any further analysis. Manual investigation also identified further exclusion criteria that lead to the removal of additional samples from the analysis. **(D)** SiRNA knock down results were analyzed as described at figure 3. Orange and blue dots show the normalized outgrowth intensity obtained for the indicated gene in one experiment at the distance that showed indicated control intensity (75%, 50%, 25%). Blue dots are outliers that were identified using Dixon's Q-Test and removed before p-value calculation using one-sample two-tailed ttest. Horizontal lines show reference control intensities. P-value results for all siRNAs with at least two replicates are summarized in figure 3B.

A

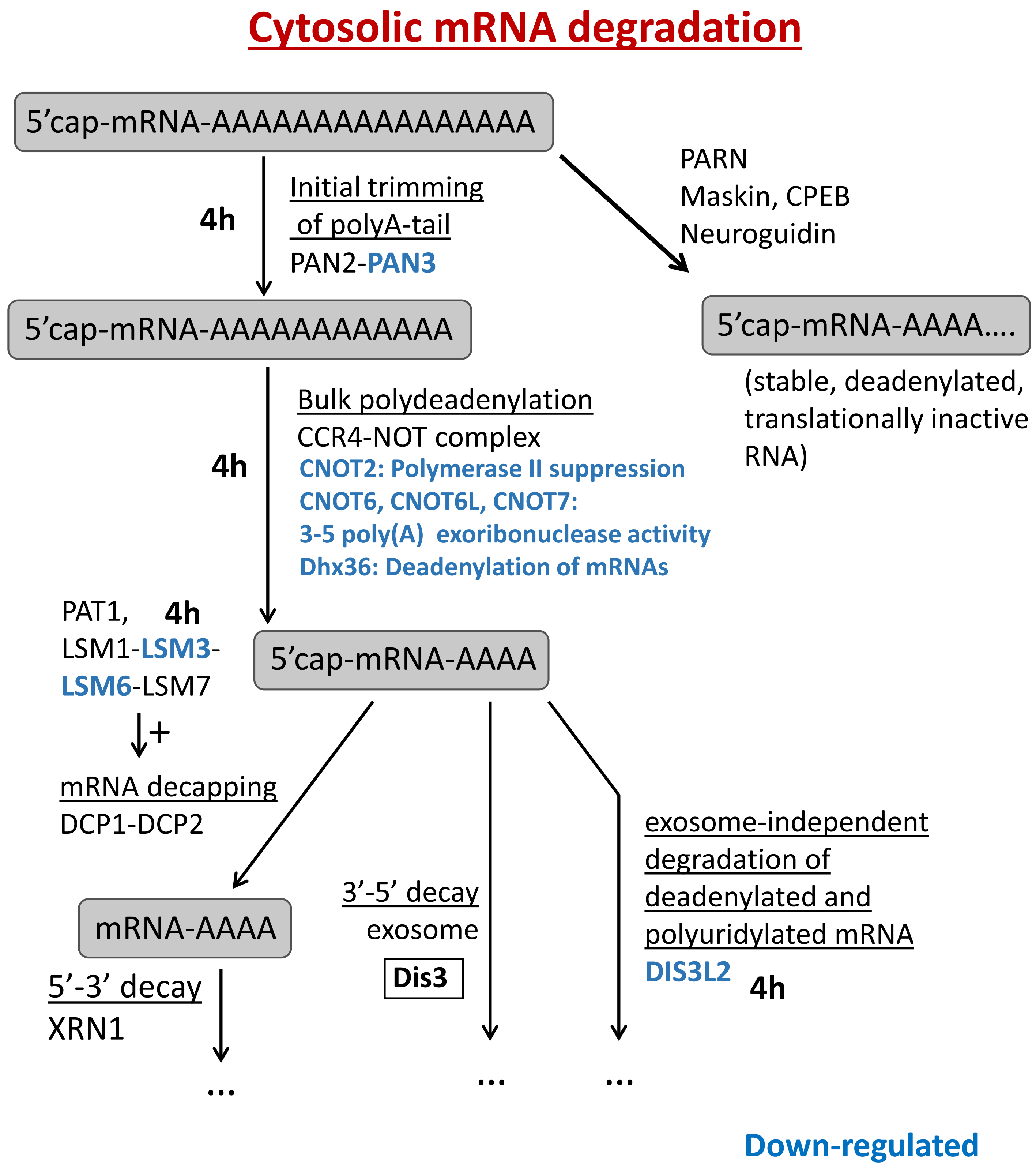

Suppl. Figure 4

B

Structural organization of mitochondria

4h

Mitochondrial transport

retrograde → anterograde

Rhot2

Rhot1

Mitochondrial fusion and fission

fission → fusion

March5, Mff

Mfn2

Mitochondrial protein import and folding

Protein import IMM

Timm44

Mtx1

Import signal removal

Immp2l

folding

Hspd1,Hspe1

Mitochondrial gene expression

mitochondrial transcription

TFAM

mitochondrial ribosomes

Mrpli3, Malsu1

mitochondrial translation

Mtif3

mitochondrial translation

Tufm

mitochondrial tRNA activation

Trmu, Vars2, Gtpbp3

Lars, Osgepl1

Mitochondrial calcium uptake

Micu3

Mitochondrial oxidative phosphorylation

Ndufb3, Cox7c

Cox7B, Cox5A, Adck3, Nudfa5, Coq10b,Ttc19

Outer membrane dynamics

Sh3glb1

Mitochondrial iron-sulfur (Fe-S) cluster assembly

Bola1

Down-regulated

Up-regulated

Suppl. Figure 4

C

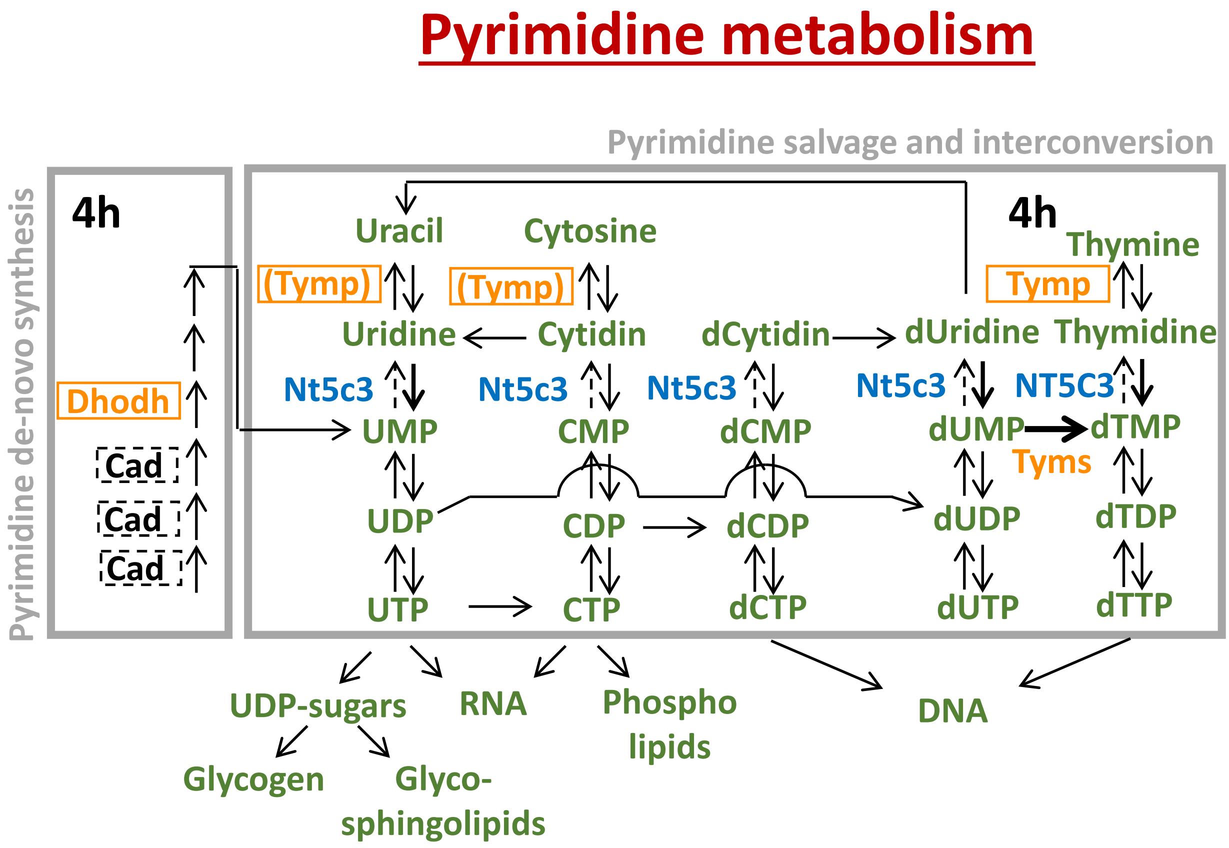

Suppl. Figure 4

D

Membrane lipid metabolism

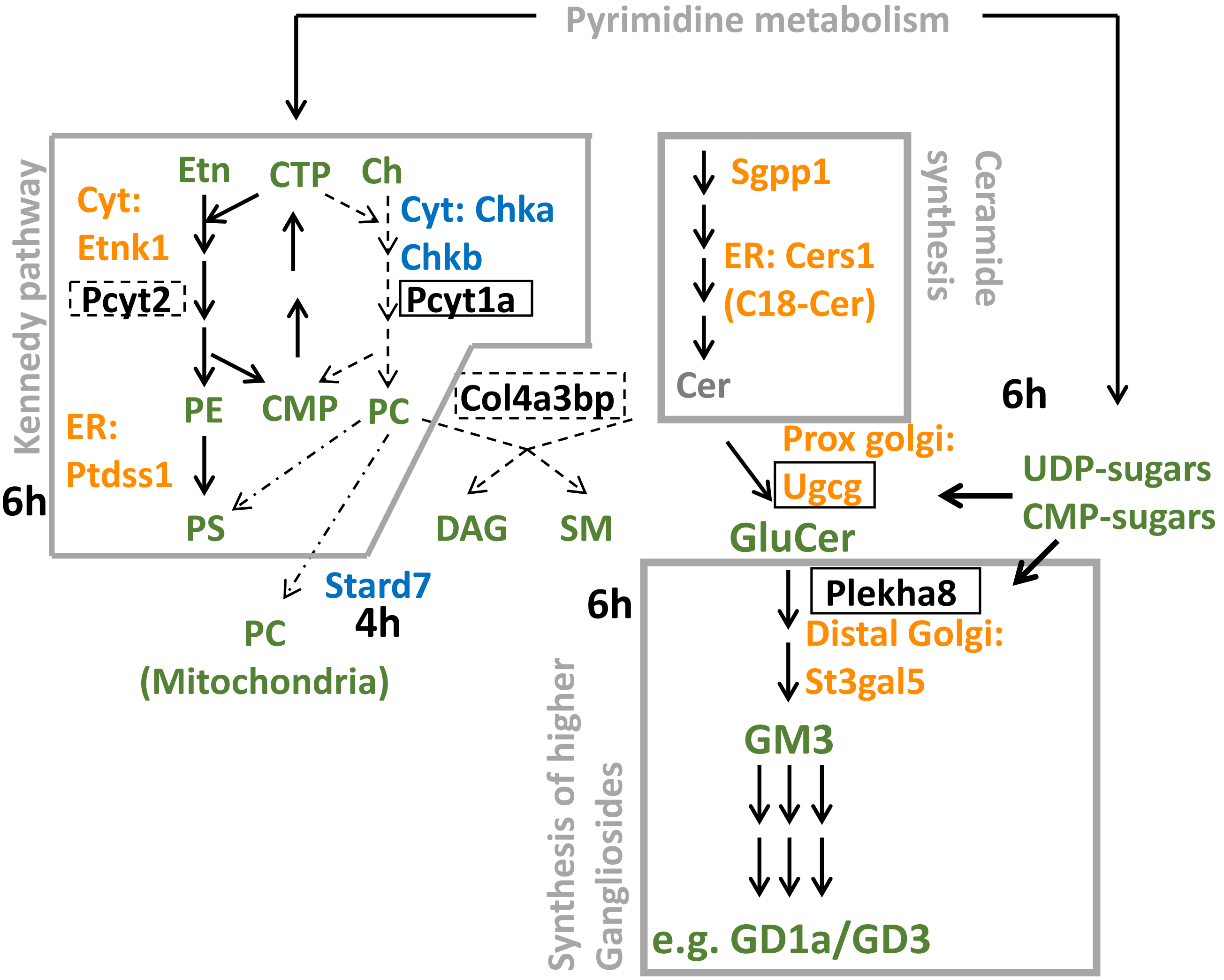

Suppl. Figure 4

E

Vesicle transport between TGN and growth cone

SCPs involved in anterograde vesicle movement from the TGN to the growth cone

Golgi vesicle transport:
6h: Ap1a1, Ap1s2, Ap3s1, Gga3, Tmed2, Rab10, Sgsm2, Bcap29, Rab6a, Golph3
8h: Ap3s1, Ap4s1, Golph3, Rab10, Tmed2, Bcap29, Rab6a
Plekha8

Microtubule based anterograde vesicle transport
6h: Ap3s1, Fyco1, Rab6a, Rab21, Snapin
8h: Ap3s1, Rab6a, Rab21
Macf1

Exocytosis:
6h: Snapin, Rala, Exoc5, Rab21, Rab2b, Rab5a, Tmem79
8h: Doc2a, Exoc1, Rab21, Tbc1d12 Vamp2, Exoc5, Dysf
Unc13b , Exoc4 , Exoc7

SCPs involved in retrograde vesicle movement from the TGN to the growth cone

Retrograde transport from endocytic pathway to Golgi
6h: Snx3, Rab9, Rab6a, Sgsm2, Pafah1b1
8h: Rab43, Rab6a, Snx3
Vps35 , Snx2

Multivesicular body genesis
Chmp6 , Hgs , Tsg101

Endosome dynamics
6h: Rab5a, Stx7, Snx10
8h: Rab5a, Ehd4

Endosomal to lysosome transport
6h: Fyco1, Rab12, Rab21 , Rnf115, Snapin
8h: Hook3, Rab21, Rnf115

Endocytosis
6h: Snx18, Rala, Fcho1, WASL, Snapin, Synj2bp, Dnm3
8h: Arf6, Hip1r, Fcho1

Recycling from endosome to plasma membrane
6h: Snx3, Linc7c, Micall2, Spast, Rab21
8h: Snx3, Rab21
Vps35 , Snx2

Suppl. Figure 4: Enzymatic reactions and gene products involved in basic cellular metabolism, structural organization of the mitochondria and vesicle transport. (A) Down-regulated genes (minimum log_2(fold change) of +/- log_2(1.3)) (blue) in cytosolic mRNA degradation (67). Genes in gray indicate other genes involved in mRNA degradation that were not differentially expressed. Genes tested in our siRNA knock down studies are framed with solid lines, if knock down changed NOG and with dashed lines otherwise. **(B)** Up- and down-regulated genes and siRNA targets involved in Structural organization of mitochondria. **(C)** Enzymatic reactions involved in pyrimidine de-novo synthesis and pyrimidine salvage and interconversion (adapted from (30) with modifications from (37)). Up- (orange) and down-regulated (blue) genes suggest a net flux of pyrimidine metabolites to pyrimidines that can be used to generate complex molecules. siRNA knock down results suggest the dependence of NOG on pyrimidine salvage, but not on pyrimidine de novo synthesis. Green: metabolites. **(D)** Enzymatic reactions and transport proteins involved in phospholipid, ceramide and ganglioside synthesis. Up- and down regulated genes as well as siRNA knock down results suggest a shift of phospholipid production from PC to PE/PS and an increase in ganglioside production. Etn: Ethanolamine, Ch: Choline, PE: Phosphatidylethanolamine, PS: Phosphatidylserine, PC: Phosphatidylcholine, Cer: Ceramide GM3/GD1a/GD3: GM3/GD1a/GD3 ganglioside, GluCer: Glucosylceramide, SM: Sphingomyelin, DAG: Diacylglycerol. **(E)** Up- and down-regulated genes in vesicle transport between the TGN and growth cone.

Suppl. table 1: Differentially expressed genes obtained after treatment of N2A cells with HU210 for 2, 4, 6 and 8hrs.

Suppl. table 2: Differentially expressed proteins obtained after treatment of N2A cells with HU210 for 5, 10 and 18hrs.

Suppl. table 3: Dynamic enrichment results of differentially expressed genes. Up- and downregulated genes at each timepoint with a minimum log_2(fold change) of +/- log_2(1.5) were separately subjected to dynamic enrichment analysis using the Molecular Biology of the Cell Ontology.

Suppl. table 4: Standard enrichment results of differentially expressed genes. Up- and downregulated genes at each timepoint with a minimum log_2(fold change) of +/- log_2(1.3) were separately subjected to standard enrichment analysis using the Molecular Biology of the Cell Ontology.

Suppl. table 5: Dynamic enrichment results of differentially expressed proteins. Up- and downregulated proteins at each timepoint were separately subjected to dynamic enrichment analysis using the Molecular Biology of the Cell Ontology.

Suppl. table 6: Standard enrichment results of differentially expressed proteins. Up- and downregulated proteins at each timepoint were separately subjected to standard enrichment analysis using the Molecular Biology of the Cell Ontology.

Suppl. table 7: Quality control of siRNA experiments. The spreadsheet documents how many replicates for each gene were considered for the statistically analysis and how many replicates were removed during our quality control assessment pipeline. Replicates that fulfilled our quality standards, but were part of an experiment where we had to remove the control replicates are also counted as removed. Similarly, we ignored all genes with only one replicate.

Suppl. table 8: Comparison of expected and observed knock down results.
